# Viral interference of nucleocytoplasmic transport

**DOI:** 10.1016/j.jbc.2025.110815

**Published:** 2025-10-14

**Authors:** Chia-Yu Chien, George W. Mobbs, Joel Ehrenkranz, André Hoelz

**Affiliations:** 1 Division of Chemistry and Chemical Engineering, California Institute of Technology, Pasadena, California, USA; 2 Howard Hughes Medical Institute, California Institute of Technology, Pasadena, California, USA

**Keywords:** nucleocytoplasmic transport, nuclear pore complex, nucleoporin, karyopherin, importin, exportin, viruses

## Abstract

Viruses have evolved diverse strategies to exploit the compartmentalized architecture of eukaryotic cells, particularly by targeting the nuclear envelope (NE) and its associated nuclear pore complexes (NPCs). The NE, composed of a double-membrane lipid bilayer, NPCs, and the nuclear lamina, establishes a physical barrier that protects genetic material in the nucleus from harmful cytosolic agents while enabling the spatial segregation of transcription and translation. This compartmentalization allows eukaryotic cells to tightly regulate gene expression, a process that requires the bidirectional shuttling of macromolecules such as transcription factors and messenger ribonucleoprotein particles across the NE. The NPC is the sole gateway to the nucleus and is essential for protein biogenesis, metabolic homeostasis, and cell survival. In this review, we examine how viruses remodel NPC architecture, hijack nucleocytoplasmic transport, and disable host innate immune responses to enhance viral replication.

Viruses are obligate intracellular parasites composed of single- or double-stranded DNA or RNA, encapsulated within a protein capsid and, in some cases, further enclosed by a lipid envelope. Unlike cellular microbes, viruses cannot replicate independently and must hijack the cellular machinery of a living host to reproduce and propagate (1, 2, 3, 4). Bacteriophages, which infect prokaryotes and archaea, replicate exclusively in the cytoplasm (5). Conversely, eukaryotic cells have evolved membrane-enclosed organelles that spatially segregate cellular processes (6). The nucleus houses genetic material and regulatory proteins involved in genome regulation, repair, and transcription, functionally segregating them from the cytoplasmic translation machinery (7, 8). This compartmentalization shields DNA from cytoplasmic stress and guards against viral genetic intrusion (9, 10, 11).

Subcellular compartmentalization imposes logistical challenges on eukaryotic cells, with ∼75% of the human proteome undergoing intracellular trafficking, and approximately one-third (>6500 unique proteins) shuttling between the cytoplasm and the nucleus (12). Protein localization is largely governed by molecular address tags that guide cargoes to dedicated transport machineries (13, 14, 15). Notably, proteins destined for the endoplasmic reticulum (ER), mitochondria, or chloroplasts are unfolded prior to membrane translocation (16). In contrast, nuclear pore complexes (NPCs), embedded at fusion sites between the inner nuclear membrane (INM) and outer nuclear membrane, facilitate transit across the nuclear envelope (NE) without disrupting the cargo protein’s tertiary or quaternary structure (17, 18, 19, 20). Human NPCs are built from 35 nucleoporins assembled in multiple copies to form a ∼120 MDa complex comprising ∼1000 polypeptides, establishing a selective gateway to the nucleus (21, 22, 23). A central transport channel (∼45–65 nm wide), occupied by a phase-separated compartment formed from intrinsically disordered phenylalanine–glycine (FG)–rich domains, establishes a size-selective permeability barrier regulating passage through the NPC (24, 25, 26). Each NPC facilitates hundreds of translocations per second, mediated by a family of ∼24 karyopherin transport factors leveraging a subcellular RAN GTPase gradient (27, 28, 29), with mean dwell times measured in the millisecond range (30, 31, 32).

Viral subversion of the nucleocytoplasmic transport machinery is essential for both propagation and pathogenesis. Viruses typically direct proteins and often their genomes to the nucleus in order to access the host's transcriptional machinery. Finally, viruses can disrupt host nucleocytoplasmic transport pathways, which are vital for mounting effective immune responses (33). In this review, we examine NPC architecture and nucleocytoplasmic transport, highlighting the diverse viral strategies that subvert these essential cellular machineries (summarized in Fig. 1). A molecular-level understanding of how viruses manipulate NPCs and the mobile nucleocytoplasmic transport machinery could inform the development of targeted therapies that block viral replication while minimizing detrimental innate immune responses.

**Figure 1:**
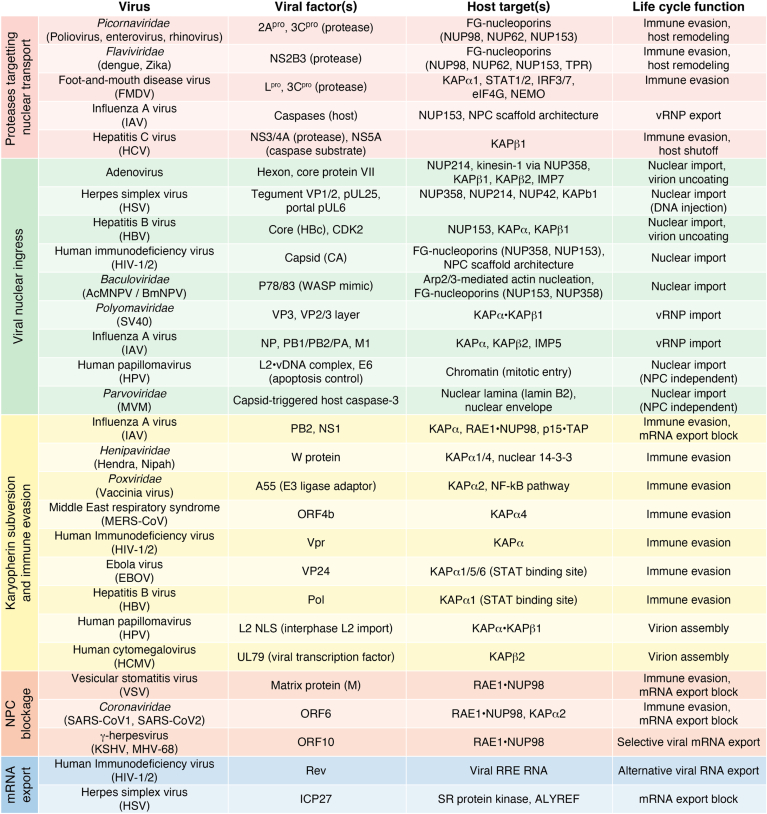
**Overview of viral strategies subverting nucleocytoplasmic transport**.

## NPC architecture

**Figure 2:**
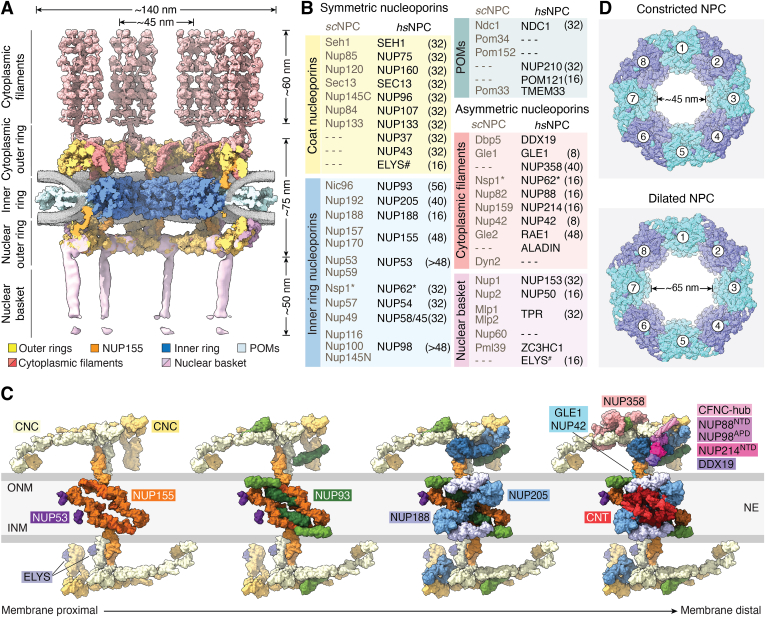
**Architecture of the human nuclear pore complex.***A*, the near-atomic composite structure of the intact human NPC, determined by quantitatively docking high-resolution nucleoporin and nucleoporin complex structures, solved by X-ray crystallography and single-particle cryo-EM, alongside a small number of homology and artificial intelligence (AI)–generated models, into an ∼12 Å cryo-ET map of the human NPC (41, 42). The nuclear envelope is visualized as a *gray* isosurface, with the individual nucleoporins and subcomplexes colored according to the legend. The cytoplasmic filaments (*red*) are modeled projecting into the cytoplasm based on available X-ray crystallography structures spanning domains flexibly attached within NUP358 homopentameric bundles in complex with their RAN, SUMO•RANGAP1, and UBC9 binding partners (41). The nuclear basket is displayed as an electron density map (*pink*) (100). *B*, nucleoporin nomenclature for the *Saccharomyces cerevisiae* (*sc*) and *Homo sapiens* (*hs*) NPCs, categorized by subcomplex. Predicted nucleoporin copy number per NPC is indicated in *parentheses*. *Asterisks* (∗) denote that NUP62 and its fungal homologs are also found in the cytoplasmic filaments. *Hash* (#) indicates that ELYS is exclusively a member of the nuclear outer ring in humans and is not symmetrically distributed. *C*, schematic *cartoon* depicting the concentric layers of the NPC, moving from the membrane proximal layer to the membrane distal position lining the central transport channel. *D*, constricted and dilated forms of the human NPC, docking high-resolution nucleoporin and nucleoporin complex structures, into ∼12 Å and ∼37 Å cryo-ET maps of the human NPC (41, 42). CFNC, cytoplasmic filament nucleoporin complex; CNC, coat nucleoporin complex; CNT, channel nucleoporin heterotrimer; cryo-ET, cryo-electron tomography; INM, inner nuclear membrane; NE, nuclear envelope; NPC, nuclear pore complex; ONM, outer nuclear membrane.

The NPC is a massive proteinaceous complex composed of a symmetric core embedded in the NE along with asymmetric structures extending into either the nucleoplasm or the cytoplasm (18, 19, 34). The symmetric core is formed from layered concentric cylinders and comprises eight spokes arranged around a central axis that exhibits pseudo–twofold symmetry in the NE plane. This symmetry is disrupted by compositional asymmetry and membrane curvature differences between the nuclear and cytoplasmic faces of the NPC (Fig. 2, *A* and *B*) (20, 24, 35, 36, 37, 38, 39, 40, 41, 42). In this section, we summarize human NPC architecture. However, detailed descriptions of near–atomic-resolution NPC structures have been reviewed recently elsewhere (43, 44, 45, 46, 47). Throughout the following sections, human nucleoporin names are reported in uppercase (*e.g.*, NUP160) and denote approximate molecular mass, whereas homologs from alternative organisms use sentence case and are preceded by a short species-specific abbreviation (*e.g.*, *sc*Nup120 for *Saccharomyces cerevisiae* [*sc*] Nup120).

### The symmetric core

The inner ring lines the central transport channel and incorporates the large scaffold nucleoporins NUP155, NUP188, NUP205, the α-helical solenoid domain of NUP93, and the coiled-coil domains of the NUP54•NUP58•NUP62 (• denote macromolecular complexes) channel nucleoporin heterotrimer (CNT), which are organized into positionally distinct subcomplexes centered on either NUP188 or NUP205 (37, 38, 40, 48, 49, 50). The inner ring is stacked radially from NUP155 (membrane-proximal) to NUP93, NUP188/205, and the CNT (Fig. 2*C*), which is an invariant architecture conserved across eukaryotes (51, 52, 53, 54, 55, 56, 57). The multivalent linker nucleoporins NUP53, NUP98, and the N-terminal region of NUP93 connect these scaffolds *via* unstructured motifs (37, 38, 40, 48, 49, 50, 58, 59). Per spoke, there are two copies of each subcomplex primarily stabilized by intraspoke linkers. The exception is NUP53, which homodimerizes at the NE to form a bridge connecting adjacent spokes (40, 60). Recent *in situ* NPC structures have revealed that inner ring constriction, triggered by ATP depletion or osmotic shifts, reduces the central channel diameter by ∼50% from ∼65 nm to 45 nm (Fig. 2*D*) (55, 57).

The outer rings sandwich the inner ring on both sides of the NE; each outer ring is composed of 16 copies of the coat nucleoporin complex (CNC; also known as the Y-shaped or NUP107–NUP160 complex). Each Y-shaped CNC is a nine-protein module, exhibiting arms formed from NUP160, NUP37, NUP75, NUP43, and SEH1, connected to a stem comprising NUP96, SEC13, NUP107, and NUP133. In the assembled NPC, these Y-complexes connect head to tail to generate two reticulated eight-membered rings (Fig. 2*C*) (35, 61, 62). In contrast to the expandable inner ring, CNCs form a relatively rigid scaffold *via* extensive subunit interactions (35, 36, 62, 63, 64). Trans-spoke CNC interactions are mediated by conserved interactions between NUP133 and NUP160 as well as by staple-like linkages formed by eight copies of NUP93 and NUP205 (37, 40, 42, 61). In human NPCs, additional NUP155 copies also bridge the inner and outer rings (36, 37, 38). Functionally, the CNC is proposed to stabilize membrane curvature, owing to its structural similarities with the coat protein complex II (COPII) vesicle coat complex (65, 66, 67, 68). In addition, it serves as a stable platform for the assembly of the asymmetric nucleoporin subcomplexes that extend into the nucleoplasm and cytoplasm.

NUP155, NUP160, and NUP133 anchor the NPC *via* amphipathic lipid packing sensor (ALPS) motifs, which detect the sparsely packed lipids characteristic of curved membrane regions (37, 69, 70, 71). In addition, transmembrane nucleoporins known as pore membrane proteins (POMs) serve as anchoring points that embed the NPC within the NE. These integral membrane proteins include NDC1, NUP210 (also called GP210), POM121, and the less well-established TMEM33, which collectively localize to the membrane region where the INM and outer nuclear membrane fuse (72, 73, 74, 75). The pore-facing soluble domain of NDC1 tethers the inner ring at the membrane by binding to the NUP155•NUP53 module and recruiting the metazoan-specific nucleoporin ALADIN (42, 76). NUP210 extends its 17 Ig-like domains into the NE lumen, where they assemble into a ring of interlinked, butterfly-shaped structures that are thought to restrict dilation of the NPC inner ring and central transport channel (Fig. 2*A*) (42, 77). Finally, while POM121’s exact location within the NPC remains unclear, it influences *de novo* NPC assembly during interphase by recruiting the membrane-interacting CNC and NUP155 to the INM. In addition, POM121 serves as a docking platform for nuclear transport factors, *via* its unstructured FG–repeat region and a suite of binding sites resembling karyopherin–cargo complexes (see *The mobile nucleocytoplasmic transport machinery* section) (78, 79).

### The asymmetric decorations

The cytoplasmic filaments consist of two functional clusters. The eponymous filaments are primarily formed by NUP358 (also called RANBP2) (80). Eight homopentameric NUP358 assemblies clamp onto stacked CNC pairs in the cytoplasmic outer ring, extending ∼60 nm into the cytoplasm (Fig. 2*A*) (41, 42). NUP358 hosts FG–repeat regions, multiple RAN-binding domains, and an E3 SUMO ligase domain that recruits SUMOylated RAN GTPase–activating protein (RANGAP1) (41, 81, 82, 83). Functionally, NUP358 acts as a docking site for import complexes and promotes export cargo release *via* RANGAP1, the GTPase-activating protein that catalyzes RAN(GTP) hydrolysis (see the *Classical nuclear import pathway* section) (84, 85). The second functional cluster consists of 16 copies of the cytoplasmic filament nucleoporin complex (CFNC) (41, 42). The CFNC is a multisubunit assembly of NUP62, NUP88, NUP214, DDX19, GLE1, NUP42, NUP98, and RAE1 organized around two RNA-binding modules: DDX19•GLE1•NUP42 and NUP88’s N-terminal β-propeller, which flexibly tethers NUP98•RAE1 (Fig. 2*C*) (41, 86, 87, 88, 89, 90, 91). These modules are connected by a central NUP88•NUP214•NUP62 heterotrimeric coiled-coil hub, which adopts an architecture reminiscent of the CNT and is also anchored by the N-terminal linker of NUP93 (41). Structural and biochemical evidence suggests there are two CFNCs per spoke in human and *Xenopus laevis* NPCs, supported by the presence of two NUP93 copies per spoke in the cytoplasmic outer rings (41, 42, 92, 93, 94). However, cryo–electron tomography (cryo-ET) reconstructions of the human NPC resolve a single CFNC hub, associated with the N-terminal β-propellers of NUP214 and NUP88, whereas density for DDX19 remains poorly defined (41, 42).

The nuclear basket is a filamentous assembly that extends ∼60 nm into the nucleus, forming a flexible net-like structure (Fig. 2*A*) (95, 96). In metazoans, the main basket protein TPR associates with NUP153, NUP50, and ZC3HC1 (97, 98, 99). Recent *in situ* cryo-ET reconstructions of focused ion beam–milled cells have provided the first low-resolution glimpse of the nuclear basket, interpreted by docking artificial intelligence–generated nucleoporin structural models from multiple species (100). These studies suggest that TPR’s coiled-coil regions form long struts projecting inward from the nuclear ring, with flexible termini clustered distally to form the basket ring. Biochemical characterization in *S. cerevisiae* indicates that nuclear basket anchoring relies on short, disordered motifs present in *sc*Nup60/NUP153, which binds the CNC component *sc*Nup85/NUP75 (101, 102). Functionally, the nuclear basket coordinates nucleocytoplasmic transport by linking transcription to mRNA processing, quality control, and export of messenger ribonucleoproteins (mRNPs) (103, 104). Newly assembled mRNPs dock at the basket, where they presumably undergo additional processing and quality-control checks before transiting the NPC (105, 106, 107). In addition, some basket nucleoporins contain FG–repeat regions or alternative binding sites for karyopherins (108, 109, 110, 111, 112), with TPR disruption impairing nuclear export without affecting import (113). The nuclear outer rings also feature ELYS, which interacts directly with the INM *via* an N-terminal β-propeller domain, whereas an adjacent α-helical solenoid domain binds the NUP160•NUP37 arm of the nuclear outer rings, with both domains required for NE localization (41, 42, 114, 115, 116). These structured domains are followed by a ∼1200-residue unstructured region containing chromatin-binding AT-hook motifs that promote nuclear localization and postmitotic NPC assembly (117, 118).

### The diffusion barrier

The NPC’s central transport channel is a phase-separated compartment filled with intrinsically disordered nucleoporin regions rich in FG–repeat motifs (119, 120, 121, 122), forming a size-selective diffusion barrier (123, 124, 125). Recently obtained NPC structures have pinpointed specific sites from which these FG–repeat regions originate (41, 42). FG–repeat regions are present in 11 nucleoporins located in either the inner ring (NUP98, POM121, and the CNT constituents NUP54, NUP58, and NUP62) or the cytoplasmic (NUP358, NUP42, NUP62, and NUP214) and nuclear (NUP153, NUP50, and TPR) outer rings. Together, these FG–repeat regions account for ∼40% of the NPC’s mass, collectively contributing >6500 FG–repeat motifs. The symmetric inner ring positions 32 CNT coiled-coil heterotrimers forming a narrow band, leading to an average FG motif concentration of ∼160 mM at the central transport channel (124). In contrast, the asymmetric FG–nucleoporins have been proposed to form a diffuse FG “cloud” (126, 127).

The permeability barrier was initially thought to block passage of macromolecules with molecular masses exceeding ∼40 kDa (∼5 nm diameter) (25, 26). However, more recent studies indicate a linear relationship between diffusion rates and mass for proteins between 30 and 150 kDa (128, 129). NPCs accommodate transport loads of ∼100 MDa per second, translocating ∼10 concurrent cargoes with mean dwell times of ∼5 ms (30, 31, 32, 130). Fluorescence microscopy and computational simulations suggest that the diffusion barrier contains subregions or “channels” with unique transport dynamics, guiding cargoes along specific pathways instead of random 3D diffusion (126, 127). Moreover, genomic deletions of FG–repeat regions in *S. cerevisiae* reveal marked redundancy, as yeast lacking up to six FG–repeat regions, including all the asymmetric FG–nucleoporins, remain viable (131). However, synthetic lethality is observed upon deletion of specific symmetric FG–nucleoporin combinations, such as all three NUP98 orthologs (*sc*Nup145N, *sc*Nup116, and *sc*Nup100) or the CNT constituents plus *sc*Nup145N, suggesting that symmetric FG–repeat regions perform essential and nonredundant roles (40, 131).

Active transport depends on transient, multivalent binding between transport receptors and FG motifs (132, 133). Structures reveal phenylalanine residues that insert into deep hydrophobic clefts present on the surface of transport factors, with adjacent glycine residues ensuring the necessary steric flexibility (134, 135, 136). These multivalent, low-affinity interactions display fast on–off kinetics to enable rapid cycles of binding and release (137, 138). Mechanistically, engineered GFP variants systematically introducing surface tryptophan and arginine residues have demonstrated that both π–π and cation–π interactions, respectively, can mediate transport through the NPC’s permeability barrier (139).

Two prevailing models explain how FG–repeat regions establish the NPC’s permeability barrier, though they are not mutually exclusive and indeed may coexist in different regions of the central transport channel. The polymer brush or virtual gating model proposes that FG–repeat regions form a steric barrier that excludes macromolecules unless their interactions with FG motifs overcome the entropic cost of polymer compression (140, 141). Evidence for brush-like behavior comes from atomic force microscopy studies on surface-tethered FG–repeats (142, 143), isolated NPCs (144, 145), and synthetic DNA origami NPC mimetics (146, 147, 148). In contrast, the selective phase or sieve model posits that cohesive FG–FG interactions create a size-selective meshwork that permits transit through local disruption of homotypic FG contacts (30, 149, 150, 151, 152).

Purified FG–repeats form size-selective hydrogels *in vitro* that exclude inert macromolecules, yet permit rapid transport factor partitioning at rates close to those observed *in vivo* (149, 150). Mechanistically, phenylalanine positions are key for self-association, as serine substitutions or the addition of aliphatic alcohols disrupt hydrogel formation (153). Accordingly, FG–repeat condensates exhibit lower critical solution temperature (LCST) behavior, where heat promotes cohesion through entropy-driven hydrophobic interactions (154, 155). For example, the GLFG repeats found in NUP98 are especially cohesive because of an abundance of π–π stacking and hydrophobic interactions, coupled with the low frequency of charged residues (156, 157). In recent years, the field has converged on the selective phase model, with rapid microfluidic dilution of *in vitro*–purified FG repeats shown to induce spherical, liquid-like droplets that mature into hydrogels (158). In addition, metabolically labeled NUP98 adopts extended configurations *in vivo*, consistent with cohesive intermolecular interactions forming a dynamic FG meshwork (159). Thus, while structurally intractable, the diffusion barrier is conceptually understood to form a phase-separated compartment spanning the central transport channel, which blocks free diffusion across the NPC.

## The mobile nucleocytoplasmic transport machinery

**Figure 3:**
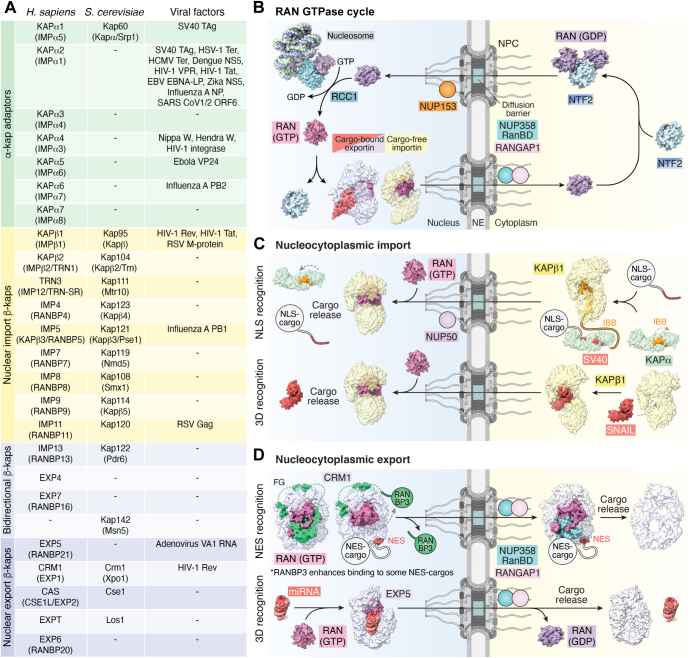
**The karyopherin mediated nuclear transport system.***A*, overview of karyopherin transport factors identified in *Homo sapiens* and *Saccharomyces cerevisiae*, alongside prominent viral interaction partners. Functionally grouped by α-karyopherin adaptors (*green*), nuclear import β-karyopherins (*yellow*), bidirectional β-karyopherins (*light blue*), and nuclear export β-karyopherins (*dark blue*). Alternative nomenclature indicated in parentheses. *B*, RAN GTPase cycle. Nuclear RAN(GTP) is incorporated into β-karyopherin complexes to trigger cargo release from importins or to promote export complex formation. Upon exiting the nucleus, the NUP358-associated GTPase-activating protein RANGAP1 stimulates RAN’s intrinsic GTP hydrolysis activity, triggering dissociation of RAN(GDP) from β-karyopherins and the release of export cargoes. Cytoplasmic NTF2 homodimers recognize free RAN(GDP) forming a heterotetrametric nuclear import complex. Upon entering the nucleus, the NPCs' nuclear basket constituent NUP153 presents four zinc finger domains capable of binding RAN(GDP). Restarting the cycle, nucleosome-bound RAN guanine exchange factor (GEF), RCC1, promotes GDP release from RAN and exchange for GTP. Structures used to generate surfaces: RCC1•RAN(nucleotide free) (PDB ID: 1I2M) (492); *dm*Rcc1•nucleosome (PDB ID: 3MVD) (493); KAPβ2•RAN(GTP) (PDB ID: 1QBK) (207); *sc*Kap122•Ran(GTP)•*sc*eIF5a (PDB ID: 6Q84); and *rn*Ntf2•*cl*Ran(GDP) (PDB ID: 1A2K) (494). *C*, nuclear import *via* classical nuclear localization signal (NLS) and 3D cargo–based recognition. Proteins exhibiting an NLS bind to KAPα, displacing the autoinhibitory importin-β-binding domain (IBB), which adopts an α-helical conformation to bind KAPβ1. Upon entering the nucleus, KAPβ1 binds to RAN(GTP) releasing the IBB, which alongside NUP50, competes with the NLS to release the cargo. In addition, many β-karyopherins directly recognize 3D structures, with nuclear RAN(GTP) triggering structural rearrangement and cargo release. Structures used to generate surfaces: *mm*Kapα2 (PDB ID: 1IAL) (495); *sc*Kapα•SV40 TAg NLS (PDB ID: 1BK6) (204); KAPβ1•KAPα2 IBB (PDB ID: 1QGK) (195); KAPβ1•RAN(GTP) kindly shared prior to publication by Gino Cingolani (209); KAPβ1•SNAIL1 (PDB ID: 3W5K) (196). *D*, nuclear export *via* helical nuclear export signal (NES), and 3D cargo–based recognition. RANBP3 primes CRM1 for NES–cargo binding. NES–cargo binds the convex face of CRM1, initiating a structural reconfiguration that displaces RANBP3. On the cytoplasmic face of the NPC, the NES–cargo•CRM1•RAN(GTP) complex binds to NUP358’s RanBD, which contacts RAN(GTP) to allosterically interfere with NES binding, whereas RANGAP1-stimulated hydrolysis of RAN(GTP) opens the superhelical fold of CRM1 to release both the NES–cargo and RAN(GDP). In addition, many export β-karyopherins recognize 3D structures such as folded domains in their RAN(GTP)-bound form, with cytoplasmic RAN turnover triggering structural rearrangement and cargo release. Structures used to generate surfaces: *sc*Crm1•*sc*Ran(GTP)•*sc*RanBP3 ternary complex (PDB ID: 3WYF) (496); *sc*Crm1•*sc*Ran(GTP)•PKI-NES (PDB ID: 3WYG) (496); *sc*CRM1•RAN(GTP)•*sc*RanBP1•PKI-NES (PDB ID: 6X2U) (497). *cl*, *Canis lupus familiaris*; *dm*, *Drosophila melanogaster*; *mm*, *Mus musculus; rn*, *Rattus norvegicus*; *sp*, *Schizosaccharomyces pombe*; *xl*, *Xenopus laevis*. GDP, guanosine-5′-diphosphate; GTP, guanosine-5′-triphosphate; IBB, importin-β binding domain; NE, nuclear envelope; NES, nuclear export signal; NLS, nuclear localization signal; NPC, nuclear pore complex; NTF2, nuclear transport factor 2; PDB, Protein Data Bank; P_i_, inorganic phosphate; RanBD, Ran binding domain; RANGAP1, Ran GTPase–activating protein 1.

The NPC is the sole conduit through the NE that enables selective, bidirectional transport of macromolecules between the nucleus and cytoplasm. Active translocation of cargoes, including proteins over >40 kDa and smaller molecules exhibiting constitutive nuclear or cytoplasmic localization, is mediated by β-karyopherin transport receptors (Fig. 3*A*) (160, 161, 162). In the following section, we discuss the discovery and structural characterization of these mobile transport factors and how they mediate transport across the NPC.

### Classical nuclear import pathway

The first nuclear localization signal (NLS) was identified in a viral protein, simian virus 40 (SV40), large tumor antigen (TAg), establishing a foundational link between viruses and nuclear transport. Approximately 95% of TAg is nuclear, but point mutations restrict it to the cytoplasm (163, 164). These mutations mapped to an unstructured basic amino acid sequence, designated the NLS, which is sufficient to redirect constitutively cytoplasmic proteins to the nucleus (165, 166, 167). Selective digitonin permeabilization of the plasma membrane revealed that cytosolic factors are required for nuclear import (168). These assays showed that NLS cargoes are initially targeted to the NE, followed by energy-dependent translocation into the nucleus. Subsequently, fractionation of *X. laevis* cytosolic extracts by anion-exchange chromatography separated two functionally distinct components: *fraction A* mediated NLS cargo recognition and docking at the NE, whereas *fraction B* supported active transport through the NPC (169). Biochemical dissection of *fraction A* revealed that its NE-targeting activity is conferred by a heterodimeric complex consisting of karyopherin-α (KAPα) and karyopherin-β1 (KAPβ1). KAPα acts as a cargo adapter, binding NLS-containing substrates through its NLS-binding domain and linking them to KAPβ1 *via* its N-terminal importin-β-binding (IBB) domain (170, 171, 172, 173). Meanwhile, KAPβ1 is essential for interacting with the FG–repeat regions that make up the diffusion barrier (172, 174, 175). *Fraction B*, which supported energy-dependent translocation of NLS cargoes into the nucleus, was found to contain the small Ras-related GTPase RAN and its cytosolic import carrier, nuclear transport factor 2 (176, 177, 178).

RAN acts as a molecular switch, cycling between GTP- and GDP-bound states, that primarily induces conformational changes in its switch 1 and switch 2 regions, thereby modulating interactions with nuclear transport proteins (81, 179). Transport directionality is maintained through an asymmetric distribution of RAN’s nucleotide states across the NE, with high concentrations of RAN(GTP) encountered in the nucleus and RAN(GDP) in the cytoplasm (Fig. 3*B*). This gradient is sustained by the compartmental segregation of RAN’s regulatory factors. The RAN guanine nucleotide exchange factor (GEF), regulator of chromosome condensation 1 (RCC1), is chromatin bound in the nucleus and promotes GDP release and GTP loading by exploiting the ∼10-fold higher intracellular GTP:GDP ratio (180, 181, 182). In contrast, RANGAP1 is tethered to the cytoplasmic face of the NPC *via* SUMOylation-dependent interactions with NUP358, where it stimulates RAN’s intrinsic GTPase activity (183).

In the nucleus, RAN(GTP) binds KAPβ1 and induces a conformational change that displaces the autoinhibitory IBB domain of KAPα, allowing it to compete with the NLS and release cargo (Fig. 3*C*). The resulting KAPβ1•RAN(GTP) complex traverses the NPC and is disassembled in the cytoplasm following GTP hydrolysis, stimulated by RANGAP1 (184). In parallel, the nuclear basket constituent NUP50 binds KAPα, accelerating cargo release and providing a platform for the formation of its cognate export complex (110, 185, 186). In contrast to its import, the released KAPα is then exported from the nucleus by forming a heterotrimeric complex with the β-karyopherin CAS and RAN(GTP) (187).

### Karyopherin diversity

The classical import pathway was foundational but covers only a small subset of nucleocytoplasmic transport events. Building upon this prototypical set, subsequent analyses guided by hydrophobicity profiling and sequence homology have identified a repertoire of seven α- and 17 β-karyopherins in humans. This includes nine import karyopherins (importins), five export karyopherins (exportins), and three bidirectional karyopherins (often termed biportins), with one α-karyopherin and 14 β-karyopherins conserved in *S. cerevisiae* (Fig. 3*A*) (162). Despite a limited number of karyopherins, this system overcomes significant logistical challenges and is capable of accommodating the vast structural and functional diversity of nuclear cargoes. Cargo recognition is achieved through diverse strategies. These include linear nonclassical NLSs, helical nuclear export signals (NESs), adapter-mediated interactions, and the direct recognition of tertiary or quaternary structures within individual proteins or macromolecular assemblies (Fig. 3, *C* and *D*) (160, 162, 188).

### β-karyopherin cargo recognition

β-karyopherins form a double-arched superhelical α-solenoid structure built from between 19 and 24 tandem Huntingtin, *E*longation factor 3, protein phosphatase 2*A*, and the lipid kinase *T*OR (HEAT) repeats, each comprising a pair of antiparallel α-helices. These helices are arranged in parallel stacks with the A and B helices lining the outer convex and inner concave surfaces, respectively (189). This arrangement confers significant conformational flexibility, with alterations to the solenoids' superhelical pitch giving rise to either S-shaped or horseshoe-shaped architectures (162).

Unstructured nonclassical NLSs, characterized by a high frequency of basic residues, have been shown to bind either the N- or C-terminal arches of various β-karyopherins (190, 191, 192). Moreover, a distinct class of NLS, characterized by an R/H/KX_2_–_5_PY consensus sequence flanked by basic or hydrophobic residues at the N terminus, dubbed the PY-NLS, binds exclusively to KAPβ2, and engages both its N- and C-terminal arches simultaneously (193, 194). Nevertheless, the structural repertoire of nonclassical NLSs remains limited and incompletely captures the full spectrum of binding modes encountered within the cell.

Beyond recognizing unstructured linear motifs, β-karyopherins can also engage folded 3D features. For example, the N-terminal IBB domain of KAPα is enveloped by the C-terminal arch of its transport receptor KAPβ1, resembling a hand gripping a handle (195). In addition, various import and export cargoes interact with both the N- and C-terminal arches of their cognate β-karyopherins. Examples include structures of importins bound to SNAIL (KAPβ1•SNAIL) (Fig. 3*C*), alternate splicing factor (TRN3•ASF), and histones H2A•H2B (IMP9•H2A•H2B), as well as exportins bound to miRNA (EXP5•RAN(GTP)•miRNA) (Fig. 3*D*), and eIF5A (EXP4•RAN(GTP)•eIF5A) (196, 197, 198, 199, 200). Throughout these structures, cargoes primarily bind along the inner concave surface of the β-karyopherin’s superhelical groove. However, a notable exception is the general exportin CRM1, which adopts a flattened, closed-ring conformation that recognizes an NES composed of a leucine-rich amphipathic α-helix on its outer convex face (Fig. 3*D*) (201, 202, 203).

### Classical NLS recognition by α-karyopherins

The α-karyopherin cargo adapters are more rigid than their β-karyopherin counterparts. Their NLS-binding domain is composed of 10 armadillo repeats, each ∼40 residues long, forming three α-helices (H1, H2, and H3) arranged into a crescent-shaped solenoid that exhibits a consistent ∼30° rotational offset (204). The outer convex surface is formed by the H1 and H2 helices, whereas H3 lines the inner concave face. Together, they create an extended groove with two distinct NLS-binding sites known as the major and minor sites. These binding sites contain parallel arrays of conserved hydrophobic tryptophan residues, three in the major site and two in the minor site, which mediate classical NLS recognition. Basic residues supplied by the NLS engage these tryptophans through hydrophobic and cation–π interactions, which are further stabilized by salt bridges formed with acidic residues lining the base of the groove. Classical NLSs can be divided into two categories: monopartite NLSs, such as that of SV40 TAg, bind to either the major site or the minor site individually. By contrast, bipartite NLSs simultaneously insert two clusters of basic residues, connected by a ∼10-residue linker, into both binding sites of a single α-karyopherin (204, 205).

### RAN(GTP) mediated cargo release and export complex formation

Despite the vast diversity of cargo-binding mechanisms observed across karyopherins, all import complexes are dismantled in the nucleus through a shared mechanism. RAN(GTP) binds to the N-terminal arch of the β-karyopherin, inducing conformational changes that promote cargo release and directly competing with bound cargoes, such as nonclassical NLSs (206, 207, 208, 209). Nuclear export complexes are likewise assembled through a common mechanism, forming an obligate heterotrimer composed of the exportin, its cargo, and RAN(GTP), which again binds at the N-terminal arch of the β-karyopherin. However, unlike import complexes, many exportins exhibit composite binding interfaces in which the cargo simultaneously contacts both the β-karyopherin and RAN(GTP), providing a structural basis for cargo selection and transport directionality. However, whereas β-karyopherins primarily mediate either nuclear import or export, several functions as bidirectional transport factors leveraging conformational rearrangement upon binding RAN(GTP) to select between import and export cargoes (Fig. 3*A*). The best characterized examples of this are IMP13 and EXP4, which form import complexes with UBC9 and SOX family transcription factors, and export complexes with eIF1a/5a, and eIF5a and SMAD3 in the absence and presence of RAN(GTP), respectively (200, 210, 211, 212, 213, 214, 215, 216).

### mRNA export

In contrast to RAN GTPase–driven nucleocytoplasmic transport, mRNA export relies on the ATPase activity of the DEAD-box helicases UAP56 and DDX19 to provide directional force for mRNP assembly, export licensing, and cytoplasmic remodeling events (217). This export mechanism involves discrete assembly and remodeling phases intricately coupled with transcription and sequential pre-mRNA processing steps, including 5′ capping, splicing, and 3′ polyadenylation (218, 219, 220, 221, 222).

Cotranscriptional maturation and licensing of pre-mRNA protects nascent transcripts from degradation and prepares them for export. Transcripts are first capped with 7-methylguanosine at their 5′ end, which subsequently associates with the cap-binding complex (CBC) (223). As transcription progresses, spliceosomes assemble on nascent intronic regions, facilitating intron excision and deposition of exon junction complexes (EJCs) (224, 225). Transcription finalizes with cleavage and polyadenylation of the 3′ end, and the recruitment of nuclear poly(A)^+^-binding proteins PABPN1 and ZC3H14, which safeguard the poly(A)^+^ tail from exonuclease degradation (226, 227). Mature mRNAs are then selectively loaded with p15•TAP export factors *via* the conserved TRanscription EXport (TREX) complex, an mRNP assembly hub composed of the THO scaffold subcomplex, UAP56, and the mRNA adapter protein ALYREF (228, 229, 230, 231). THO•UAP56 binds nascent pre-mRNAs during transcription elongation (232, 233, 234). Initially, ALYREF associates with EJCs, CBC, and poly(A)^+^ tails; it then recruits UAP56 to create a composite interface connecting the THO complex to the mRNP (224, 228, 229, 234, 235, 236, 237, 238). Recent structural studies demonstrate that the human TREX core assembles into a flexible ∼2 MDa module, comprising a central THO hub flanked by two UAP56 helicases, presenting multiple exposed binding sites for mRNA and TAP (239). In addition, nuclear mRNPs adopt compact globular conformations (∼20–30 nm), which are densely coated with TREX complexes (240). The ATPase activity of UAP56 is proposed to mediate mRNP compaction by remodeling mRNA–protein interactions, displacing the THO complex and stabilizing ALYREF binding (240, 241, 242, 243). Remodeled mRNPs dock at the nuclear basket *via* TREX-2, which displaces UAP56 and coincides with p15•TAP loading, enabling translocation across the NPC (244, 245).

Structurally, p15 and TAP resemble the RAN(GDP) importer nuclear transport factor 2. Passage through the NPC is mediated by transient FG–repeat interactions with hydrophobic pockets on TAP and the heterodimeric p15•TAP interface (136, 246, 247, 248, 249). Crucially, p15•TAP loading serves a quality control function, ensuring only fully processed transcripts are exported. This key licensing step is circumvented by viral RNA elements, such as the HIV-1 constitutive transport element, which binds TAP with high affinity, resulting in the aberrant export of unspliced pre-mRNAs (250, 251). Single-molecule fluorescence microscopy has revealed that mRNP transit through the NPC consists of three kinetic phases: a slow docking phase (∼80 ms), rapid translocation through the central transport channel (∼5–20 ms), and slow release into the cytoplasm (∼80 ms) (252). However, the precise molecular mechanisms underlying these stages remain unclear. At the cytoplasmic face, the helicase activity of DDX19 remodels mRNPs, releasing p15•TAP and ZC3H14 and preventing futile reimport (90, 91, 253, 254, 255). Cryo-ET structures of the human NPC localize DDX19 to the cytoplasmic outer rings (41). Arranged like a crane hovering over the central transport channel, binding NUP214's N-terminal β-propeller domain. Alongside NUP214, DDX19 also interacts with GLE1 and single-stranded RNAs, with its catalytic cycle dynamically regulated by RNA- and GLE1-dependent activation, counterbalanced by NUP214-mediated inhibition (86, 87, 90, 91).

## Proteases targeting nucleocytoplasmic transport

**Figure 4:**
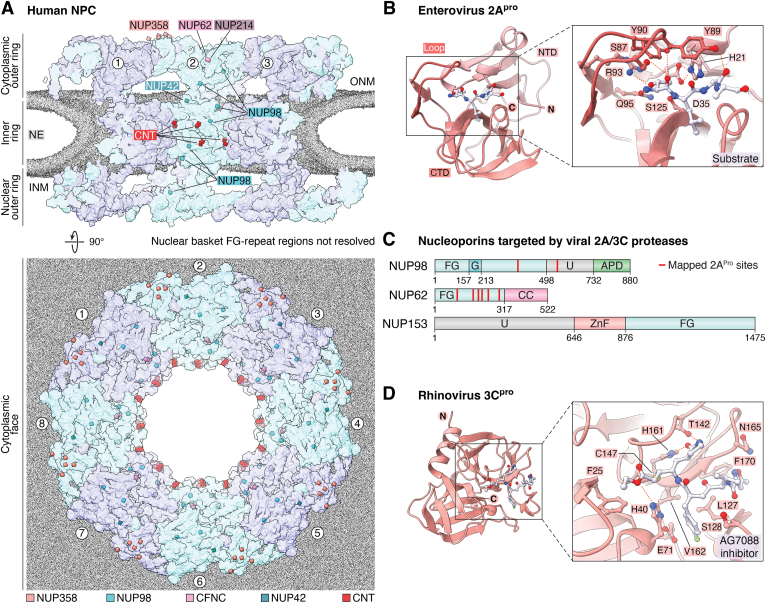
**The FG–repeat diffusion barrier is a hot spot for viral proteases.***A*, the near-atomic composite structure of the intact human NPC (41, 42), the nuclear envelope is depicted as a gray isosurface, eightfold symmetric spokes are represented in *transparent cartoon surface* format (alternating *cyan/purple*). Nucleoporin locations on the NPC scaffold where FG–repeat regions project from are colored as indicated in the legend. *B*, crystal structure of inactive enterovirus-A71 2A^pro^ in complex with a canonical substrate peptide derived from the VP1-2A cleavage junction (PDB ID: 4FVD) (498). *C*, domain architectures of NUP98, NUP62, and NUP153, human FG–nucleoporins targeted by viral 2A^pro^ and 3C^pro^, *red lines* indicate biochemically mapped 2A^pro^ cleavage sites (264, 267). Domain abbreviations: APD, autoproteolytic domain; CC, coiled-coil domain; FG, FG–repeat region; G, GLEBS domain; ZnF, zinc finger domain; U, unstructured. *D*, crystal structure of rhinovirus-A2 3C^pro^ bound to the AG7088 inhibitor (PDB ID: 1CQQ) (499). CFNC, cytoplasmic filament nucleoporin complex; cryo-ET, cryo-electron tomography; FG, phenylalanine–glycine; INM, inner nuclear membrane; NE, nuclear envelope; NPC, nuclear pore complex; ONM, outer nuclear membrane; PDB, Protein Data Bank.

Viruses from diverse families have evolved distinct mechanisms to subvert nucleocytoplasmic transport, often through direct disruption of the NPC. A prominent strategy employed by many single-stranded RNA viruses involves viral-encoded proteases, which cleave specific nucleoporins bearing FG–repeat regions that line the central transport channel (Fig. 4*A*; see *The diffusion barrier* section) (256, 257). Proteolytic cleavage compromises the structural integrity of the NPC, impairing bidirectional transport across the NE. In addition, viral proteases often exhibit broad promiscuity. For example, computational predictions indicate that ∼50 human proteins, including factors involved in mRNA processing, DNA repair, and apoptosis, are susceptible to cleavage by the severe acute respiratory syndrome coronavirus 2 (SARS-CoV2) main 3C-like protease (258).

### Viral proteases disrupting NPC architecture

Positive-sense single-stranded RNA viral genomes are translated into large polyproteins in the cytoplasm, which are subsequently cleaved by viral proteases into discrete functional subunits (257). Despite replicating exclusively in the cytoplasm, several viral proteases also target the NPC. Early studies investigating poliovirus and rhinovirus infections in HeLa cells revealed compromised NPC function, indicated by ectopic cytoplasmic accumulation of nuclear proteins and reciprocal inhibition of nuclear mRNA export (259, 260). Import assays using infected digitonin-permeabilized HeLa cells demonstrated that protein mislocalization arises from widespread defects impacting both classical nuclear import and alternative import routes mediated by KAPβ2 (261). In both cases, the observed transport block was traced to the selective degradation of two FG–nucleoporins: NUP153, a component of the nuclear basket, and NUP62, a constituent of the CNT and CFNC complexes, located at the central transport channel and cytoplasmic filaments, respectively.

Subsequent studies identified the 2A protease (2A^pro^) as the causative agent for this bulk nuclear transport defect throughout the Picornaviridae family (including poliovirus, coxsackievirus, rhinovirus, cardiovirus, and enteroviruses) (Fig. 4*B*) (261, 262, 263). In addition to targeting NUP62, both coxsackievirus and poliovirus 2A^pro^ cleave NUP98, the most abundant nucleoporin accounting for approximately one-third of the FG motifs at the NPC, excising its entire N-terminal FG–repeat region (262, 264). Coincidentally, the NUP98 FG–repeat region also contains the GLEBS domain, which anchors the cytoplasmic filament constituent RAE1, a nucleoporin implicated in the export of both mRNA and pre-60S ribosomal subunits (88, 265, 266).

Curiously, of the three nucleoporins targeted by 2A^pro^, NUP98 is cleaved most rapidly, with significant activity observed within 30 min of infection, whereas NUP62 and NUP153 are targeted later during active viral replication (259, 261). N-terminal sequencing of *in vitro*–treated NUP62 by Edman degradation revealed that rhinovirus 2A^pro^ selectively cleaves at six glycine residues located between the N-terminal FG–repeat region and the C-terminal coiled-coil domain, with glycine-to-alanine substitutions abolishing proteolysis (Fig. 4*C*) (267). In contrast, poliovirus 2A^pro^ exhibits markedly different substrate specificity, retaining a longer fragment of the NUP62 FG–repeat region. Although the precise functional roles of individual FG–nucleoporins remain to be elucidated, the differential cleavage patterns observed across six rhinovirus 2A^pro^ genotypes correlate with distinctive symptoms. Rapidly acting 2A^pro^ from the human rhinovirus B (HRV-B) family more efficiently disrupts karyopherin-mediated translocation across the NPC and is proposed to suppress host innate immune responses. In contrast, the HRV-A and HRV-C families permit partial immune system activation and are associated with heightened inflammatory symptoms (268, 269).

Beyond the 2A^pro^ family, two additional classes of viral proteases target the NPC. Among these, rhinovirus 3C protease (3C^pro^) specifically cleaves the C-terminal FG–repeat region of NUP153 (Fig. 4*D*) (270). This discovery provided fresh insight into the temporal coordination of NPC disruption during viral infection. Notably, 2A^pro^ cleaves NUP98 early (∼0.5–3 h postinfection [PI]), followed by NUP62 at the later stages (∼12–24 h PI), whereas 3C^pro^-mediated cleavage of NUP153 occurs at an intermediate time point (∼9 h PI). These distinct proteolytic events correlate with the mislocalization of specific nuclear factors, suggesting temporal coordination of host cell disruption. For example, 3C^pro^ redistributes the serine/arginine-rich splicing factor SC35, an essential regulator of alternative splicing, from nuclear speckles to the nucleoplasm (271). Similarly, nucleolin, a nucleolar protein that links pre-rRNA transcription with ribosome assembly, is displaced to the nucleoplasm (272). Together, these findings suggest that 3C^pro^ disrupts cellular homeostasis by impairing the regulation of alternative splicing and ribosome biogenesis. Alternatively, the fast-acting 2A^pro^ cleaves NUP98, mislocalizing the hnRNP-A1 and hnRNP-C1/C2 pre-mRNA processing factors, functionally akin to "RNA nucleosomes," from the nucleus to the cytoplasm, disrupting cotranscriptional mRNA processing (273). Finally, NS2B3 serine/threonine proteases encoded by the dengue and zika positive-sense single-stranded RNA *Flaviviruses* also target a similar subset of FG–nucleoporins (274). However, in addition to cleaving NUP62, NUP98, and NUP153, zika virus NS2B3 also targets TPR, the dominant scaffold component of the nuclear basket, proteolytically removing its C-terminal FG–repeat region. In contrast to the selective activity of picornaviral 2A^pro^ and 3C^pro^, NS2B3 also induces significant morphological defects at the NE.

### Host pathways targeted by viral proteases

Direct cleavage of nucleoporins is not the sole mechanism by which viral proteases impair bidirectional transport. An illustrative example is the foot-and-mouth disease virus (FMDV) leader protease (L^pro^), a papain-like protease that, unlike 2A^pro^ or 3C^pro^, does not cleave nucleoporins. Instead, L^pro^ localizes to the nucleus and cleaves the transcription factors involved in interferon (IFN)-β signaling, such as the IFN regulatory factors IRF3, IRF7, and the heterodimeric STAT1/2 (275). Cleavage of STAT1/2 impairs the expression of interferon-stimulated genes (ISG), which ordinarily serve as the vanguard of the host’s innate immune response, enhancing the activation of local immune cells and promoting an antiviral state in neighboring uninfected cells (276, 277, 278). In addition, FMDV L^pro^ proteolyzes the translation initiation factor eIF4G, halting cap-dependent translation and triggering shutdown of host protein biogenesis (279). Another example is the poliovirus 3CD polyprotein (3C^pro^-3D polymerase precursor). Outside NUP153, poliovirus 3C^pro^ cleaves the TATA-binding protein and several other transcription factors in the nucleus, triggering rapid inhibition of host mRNA transcription (280). Notably, 3C^pro^ lacks an NLS of its own and is imported exclusively as part of the 3CD polyprotein, co-opting the import capacity of the 3D polymerase domain. Following nuclear entry, the polyprotein undergoes autoproteolytic maturation, releasing active 3C^pro^, which subsequently degrades TATA-binding protein silencing transcription through inhibition of all three host RNA polymerases (281).

Beyond virally encoded proteases, many viruses also redirect the activity of host enzymes, notably the cysteine-aspartic protease (caspase) family, which orchestrates apoptosis. As premature host cell death undermines productive viral replication, viruses have evolved diverse mechanisms to suppress caspase activation and inhibit apoptotic signaling. These include downregulating death receptor expression and preventing mitochondrial release of cytochrome-*C*, a key apoptotic trigger (282). For example, human papillomavirus type 16 (HPV-16) encodes the oncogenic protein E6, which stabilizes procaspase-8, thereby preventing its autoproteolysis and activation, effectively blocking apoptosis (283). Conversely, certain viruses exploit caspase activity to advance their replication cycle. Influenza A virus (IAV), for example, utilizes caspase-mediated cleavage of NUP153 that has been proposed to induce NPC dilation, facilitating the nuclear export of viral ribonucleoprotein (vRNP) complexes (284). Moreover, several viral proteins are directly processed by caspases, including hepatitis C virus (HCV) NS5A and the IAV NP, which are cleaved to produce truncated cytosolic forms (285, 286).

### Viral proteases targeting karyopherins

Macromolecular cargoes rely on the karyopherin transport machinery to regulate their subcellular location and functionality (see *The mobile nucleocytoplasmic transport machinery* section). Several viruses utilize proteolytic virulence factors to directly cleave karyopherins, leading to reduced nuclear import activity. RNAi screening of HCV–host interactions in a Sendai virus infection model has identified ∼50 genes involved in mounting an effective innate immune response, including karyopherins and the cytoplasmic filament constituent NUP214 (287). Among these, silencing KAPβ1 yields the most pronounced suppression of ISG expression. Mechanistically, the HCV NS3/4A protease cleaves the C-terminal HEAT repeat of KAPβ1. Several cargoes, including the KAPα IBB domain, bind the C-terminal arch of KAPβ1 (195). This results in broad impairment of the classical nuclear import pathway, leading to reduced nuclear localization of key antiviral effectors, such as IRF3, NF-κB, and STAT1, a phenotype rescued upon the introduction of cleavage-resistant KAPβ1 mutants (287).

In addition to directly targeting STAT transcription factors *via* L^pro^, FMDV also employs 3C^pro^ to antagonize the Janus kinase (JAK)–signal transducer and activator of transcription (STAT) signaling pathway by degrading KAPα import adapters (288). HeLa cells overexpressing 3C^pro^ and treated with IFNβ exhibit inhibited IFN-stimulated response element promoter activity, resulting in the depletion of ISG transcripts. 3C^pro^ preferentially degrades KAPα1 and the NF-κB essential modulator (NEMO), specifically preventing nuclear import of STAT1/STAT2 and resulting in the near-complete blockade of innate immune signaling (288, 289). Likewise, Seneca Valley virus 3C^pro^ also degrades KAPα1 to counteract the JAK–STAT pathway (290).

## Viral ingress: Breaching the NE

**Figure 5:**
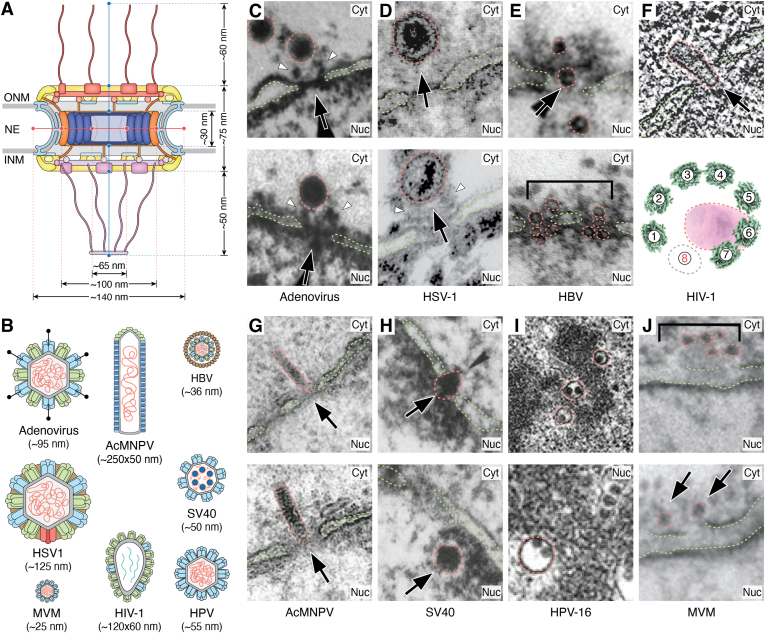
**Viral entry to the nucleus.** To scale cutaway *cartoon views* of (*A*) the dilated NPC’s architecture and (*B*) viral capsids. (*C*–*H*) EM visualization of viral capsids (*red*) traversing the nuclear envelope (NE; *green*). *C*, thin section EM of digitonin-permeabilized HeLa cells, supplemented with purified partially uncoated adenovirus capsids, NPC densities corresponding to the cytoplasmic filaments are indicated (*white arrows*) (304). *D*, thin section EM of herpes simplex virus-1 (HSV-1) capsids associated with the cytoplasmic face of the NPC. HSV-1 infected Vero cells, DNA loaded capsid approaching NPC (*top*). *In vitro*–purified nuclei incubated with uncoated HSV-1 capsids (*bottom*), NPC densities corresponding to the cytoplasmic filaments are visualized contacting the capsid vertices (*white arrows*) (309). *E*, thin section EM of *Xenopus laevis* oocytes microinjected with purified hepatitis B virus (HBV) capsids demonstrates that the capsid can translocate the NPC intact (326, 500). *F*, slice through a cryo-ET reconstruction of SupT1-R5 cells infected with HIV-1 (*top*) (344). Template matching of an NPC’s inner ring derived from cryo-ET reconstructions of human monocyte–derived macrophages infected with HIV-1 (*bottom*) demonstrates that passage of HIV-1 capsid can induce localized disruption of the NPC’s eightfold symmetry (345). *G*, thin section EM of LLC-PK1 porcine kidney cells infected with autographa californica multiple nucleopolyhedrovirus (AcMNPV) crossing the NPC (349). *H*, thin section EM of TC7 monkey kidney epithelial cells coinjected with simian virus 40 (SV40) and mAb414, an antibody that targets FG–repeat regions on NUP358, NUP214, NUP62, and NUP153. SV40 capsids visualized translocating the NPC (*top*) and postimport contacting the nuclear lamina (*bottom*) (501). *I*, thin section EM of human papillomavirus-16 (HPV-16)–infected HeLa cells. Visualizing intact HPV-16 capsids located in the cytoplasm (*top*) and nucleus (*bottom*), in premitotic and postmitotic cells, respectively (388). *J*, thin section EM of *X*. *laevis* oocytes injected with minute virus of mice (MVM) displays NE ruptures (395). cryo-ET, cryo-electron tomography; Cyt, cytoplasm; INM, inner nuclear membrane; NE, nuclear envelope; Nuc, nucleus; NPC, nuclear pore complex; ONM, outer nuclear membrane. *Panels* (*C*, *D*, *E*, *F*, and *J*) are adapted from the indicated refs. CC BY 4.0 (https://creativecommons.org/licenses/by/4.0/). *Panel* (*E*) is adapted from (326). Copyright (2003) National Academy of Sciences. *Panels* (*G*–*I*) are adapted from the indicated references. Used with permission of the American Society for Microbiology; permission conveyed through Copyright Clearance Center, Inc.

The majority of DNA viruses replicate within the nucleus, where they co-opt the host cell’s DNA replication machinery (11). A notable exception is the Poxviridae family (*e.g.*, smallpox, vaccinia, monkeypox), which replicates exclusively in the cytoplasm by assembling membrane-enclosed, ER-associated “replication factories” that concentrate viral enzymes, including a DNA polymerase and the helicase–primase complex (291). In contrast, relatively few RNA viruses replicate in the nucleus. Notable exceptions include members of the Orthomyxoviridae (*e.g.*, IAV) and Bornaviridae families (292, 293). In addition, some viruses employ hybrid replication strategies. For example, retroviruses (*e.g.*, HIV-1) rely on host factors to reverse transcribe their RNA genome into DNA, which is subsequently integrated into the host’s genome (294). Conversely, Hepadnaviridae (*e.g.*, hepatitis B virus [HBV]) uses a complementary positive sense RNA intermediate to encode a partially double-stranded DNA genome that is converted into covalently closed circular DNA in the nucleus (295, 296). Regardless of their replication strategy, viruses encounter the NE as a physical barrier during interphase, with passive diffusion across the NPC’s permeability barrier typically limited to macromolecules smaller than ∼5 nm in radius (see *The diffusion barrier* section) (25, 26). Thus, even the smallest viral capsids, such as porcine circovirus, which measures ∼18 nm in diameter, exceed the passive diffusion limit (297). Moreover, viral capsids often match or exceed the diameter of the NPC’s central transport channel, which measures ∼65 nm when dilated (Fig. 5, *A* and *B*) (40, 42). This section describes the distinct strategies viruses have evolved to breach the NPC and gain access to the nucleus.

### Capsid docking and genome translocation through the NPC

Large DNA viruses have been visualized by EM docking at the NPC, where biochemical analyses suggest that capsid–nucleoporin interactions facilitate genome translocation into the nucleus (11). Adenoviruses are nonenveloped double-stranded DNA viruses encased within an icosahedral capsid ∼90 nm in diameter (298). In the cytoplasm, the virion is partially disassembled, triggering uncoating and release of the outer capsid components (299). Upon reaching the NPC, the hexon coat protein binds exclusively to the N-terminal β-propeller domain of NUP214 (Fig. 5*C*) (300, 301). The E3 ubiquitin ligase mind bomb 1, identified in genome-wide RNAi screens, is an essential host licensing factor for adenoviral capsid disassembly at the NE (302). Loss of mind bomb 1 function, either through depletion or ligase-inactivating mutations, results in the accumulation of genome-loaded capsids docked at the NPC. Concurrent with viral capsid association at the NPC, the microtubule motor kinesin-1 is recruited to drive capsid disassembly. Dismantling involves direct capsid interactions with kinesin-1 light chains, KLC1 and KLC2, and an indirect interaction mediated by NUP358, which subsequently recruits and activates the kinesin-1 heavy chain, KIF5C (303). Notably, capsid disassembly coincides with NPC damage, as evidenced by elevated NE permeability, and the colocalization of nucleoporins and capsid fragments with kinesin-1 light chains at the cell periphery. Collectively, these observations support a model in which the capsid forms a stable anchoring interaction at the NPC, which recruits motor proteins to mechanically pull apart the capsid.

Upon capsid disassembly, the viral DNA (vDNA) genome traverses the NPC *via* a poorly understood mechanism involving several host factors, including histone H1, heat shock protein 70, and the nuclear import karyopherins KAPβ1, KAPβ2, and IMP7 (300, 304, 305). Given the involvement of karyopherins, the adenoviral major core protein VII has been proposed to serve as a protein adapter (306), as it remains associated with the viral genome following capsid disassembly and has been shown to translocate across the NPC in digitonin-permeabilized HeLa cells while bound to KAPβ2 (305).

Another example is the Herpesviridae family, which includes the causative agents of herpes simplex, varicella (chickenpox), herpes zoster (shingles), and infectious mononucleosis. Herpes simplex virus-1 (HSV-1) is an enveloped, double-stranded DNA virus with an icosahedral capsid measuring ∼125 nm in diameter (307). In common with adenoviruses, HSV-1 capsids bind to the cytoplasmic face of the NPC, which orients the vertices relative to the NPC’s central transport channel (Fig. 5*D*) (308, 309, 310, 311). A combination of genetic and biochemical antibody–blocking assays has demonstrated that both cytoplasmic filament nucleoporins (NUP358, NUP214, and NUP42) and transport factors (KAPβ1 and RAN) are essential for viral docking at the NPC (309, 312, 313). Viral factors involved in this process include the outer tegument proteins VP1/2 and the minor capsid protein pUL25, the latter of which has been shown to interact with NUP214 and NUP42 (309, 312). Unlike adenoviruses, which rely on mechanical disruption, the HSV-1 capsid remains docked at the NPC throughout DNA injection (308). EM approaches have determined that DNA release occurs through the capsid portal structure, a 12-membered ring formed by pUL6 and located on the vertex facing the NPC (314, 315). Owing to genome packaging features shared with bacteriophages, herpesviruses are proposed to employ an analogous pressure-driven genome expulsion mechanism (316). Evidence supporting this model includes the observation that the last region of DNA to be packaged is the first to be injected and subsequently transcribed by the host cell (317). Meanwhile, *in vitro* experiments have demonstrated that supplementing cytosolic extracts with 30% (w/w) PEG 8000 generates an external osmotic pressure sufficient to counteract the internal pressure within the HSV-1 capsid, suppressing genome ejection into reconstituted nuclei (318).

### Passage of intact capsids across the NPC

Certain DNA viruses and retroviruses possess capsids that are sufficiently small to traverse the NPC intact, utilizing the central transport channel in a manner analogous to large endogenous cargoes. HBV is an enveloped, partially double-stranded DNA virus ∼45 nm in diameter. Its genome is encapsulated within an ∼36 nm icosahedral capsid, formed from 240 copies of a single protein, the HBV core (HBc) (319). Post-translational modifications play a pivotal role in regulating key lifecycle events, particularly nuclear ingress (320, 321, 322). The C-terminal arginine-rich domain of HBc contains multiple phosphorylation sites, which are targeted by host kinases, such as PKC, serine–arginine protein kinase 1, and serine–arginine protein kinase 2 (320, 323, 324). Single-molecule fluorescence microscopy experiments tracking HBV capsids lacking this C-terminal domain reveal prolonged residence at the NPC, located in peripheral nonproductive positions ∼44 nm from the midpoint of the central transport channel (325). Upon reaching the NPC, intact HBV capsids leverage host karyopherins to traverse the NPC (Fig. 5*E*) (326). However, prior to karyopherin binding, the intrinsically disordered C-terminal domain, previously sequestered within the capsid, is phosphorylated, then protrudes through pores located at the quasi-sixfold axes between capsid vertices, exposing ∼30 distinct binding sites for KAPα and KAPβ1 (327). Each of these disordered regions harbors both classical and nonclassical NLSs, two putative NESs, and is interspersed by three conserved phosphorylation sites (322, 328). A recent cryo-EM structure of intact HBV capsids shows that KAPα binds to a monopartite NLS at the extreme C terminus of HBc (322). This finding suggests that the mechanistic role of HBc phosphorylation is tied to compacting the unstructured C-terminal region, drawing it closer to the capsid’s surface to maintain favorable dimensions for NPC translocation.

Upon traversing the NPC, the HBV capsid is retained at the nuclear basket by interactions with NUP153, where it is transiently dismantled, releasing HBc dimers and vDNA into the nucleus (329, 330). Curiously, this NPC “doorstop” selectively disassembles DNA-loaded capsids *via* NUP153 recognition, with RNA-loaded mimetics retained undamaged at the nuclear face (330). Although the mechanism of HBV capsid disassembly remains unclear, inhibiting the capsid-embedded cyclin-dependent kinase 2 (CDK2) reduces nuclear circular DNA formation, suggesting that CDK2-mediated phosphorylation contributes to disassembly (331). In addition, all-atom molecular dynamics simulations have revealed discrete weak points within the capsid that may be disrupted by mechanical force, with the NPC potentially serving as the requisite anchoring site (332).

The infectious retrovirus HIV-1 core exhibits a cone-shaped capsid ∼120 nm long and 50 to 60 nm wide, built from 250 copies of the capsid protein (CA), arranged in a lattice comprised of both hexamers and pentamers. Until recently, prevailing models held that the HIV-1 capsid must either disassemble in the cytoplasm to let the reverse-transcribed DNA enter the nucleus or, alternatively, traverse the NPC utilizing exposed NLSs together with direct contacts between the capsid and FG–repeat nucleoporins such as NUP358 and NUP153 (333, 334, 335, 336, 337, 338). Accordingly, silencing either NUP153 or NUP358 significantly reduces HIV-1 import, underscoring their essential roles in capsid docking and translocation (339, 340, 341). Recent studies show that although individual HIV-1 capsid components fail to partition into reconstituted NUP98 hydrogels that mimic the NPC’s diffusion barrier, fully assembled capsids can bind directly to FG–repeat domains, bypassing the need for transport factors (342, 343). This observation is consistent with emerging cryo-ET studies visualizing HIV-1 capsids traversing the NPC intact, demonstrating that the ∼60 nm wide capsid can be accommodated by dilation of the central transport channel (344). Although in some instances, localized “cracking” of the NPC’s inner ring scaffold has been observed, potentially facilitating capsid passage into the nucleus (Fig. 5*F*) (345). However, the temporal relationship between NPC cracking and either HIV-1 capsid engagement or translocation remains to be determined. Minimal DNA-origami NPC mimetics, featuring a ∼60 nm wide channel functionalized with vertical layers of NUP358, NUP62, and NUP153, incorporating the C-terminal cyclophilin domain of NUP358 and the FG–repeat regions of NUP62 and NUP153, are sufficient to promote HIV-1 capsid translocation (346). Owing to its higher capsid affinity, NUP153 is thought to drive stepwise translocation across the NPC: whereby NUP358 mediates the initial docking, NUP62 acts as a barrier that selects permissive capsid orientations, and NUP153 guides the capsid through the central channel.

Larger viruses are also capable of translocating through the NPC, such as the enveloped double-stranded DNA baculovirus, *Autographa californica* multiple nucleopolyhedrovirus, which possesses an elongated, rod-shaped capsid measuring ∼300 × 25 × 25 nm (347, 348). In contrast to karyopherin-mediated selective import or intrinsic affinity for FG–nucleoporins, baculoviruses employ a brute force strategy. Mimicking the host Wiskott–Aldrich syndrome protein, viral capsid protein P78/83 activates the actin-related protein 2/3 complex, a key nucleation factor promoting the formation of branched actin filaments (349, 350). In this case, actin polymerization is morphologically confined to one end of the capsid to form a characteristic “comet” tail, which generates the propulsive force required for cytosolic transit and NPC translocation. Experiments in digitonin-permeabilized HeLa cells lacking cytoplasmic factors (*e.g.*, karyopherins) showed that baculoviral capsids cross the NPC independent of host transport machineries (Fig. 5*G*), *via* an actin-driven pathway, that can be inactivated by actin-polymerization inhibitors (351). In the closely related *Bombyx mori* nucleopolyhedrovirus, NUP358 and NUP153 depletion leads to the cytoplasmic accumulation of capsids (352). Furthermore, targeted deletion of the NUP153 FG–repeat region yields a similar phenotype, suggesting that in common with HIV-1, baculoviral capsids also depend on permissive FG–repeat interactions.

### Partial capsid disassembly and import of subviral components

Beyond docking and translocation of intact capsids at the NPC, several viruses undergo cytoplasmic disassembly and remodeling to repackage their genomes, forming vRNP complexes capable of nuclear import. Examples include the small double-stranded DNA viruses from the Polyomaviridae (*e.g.*, SV40 and John Cunningham virus) (353) and Dependoparvoviridae (*e.g.*, adeno-associated virus) families (354) and the large enveloped negative-sense single-stranded RNA Bornaviridae (*e.g.*, borna disease virus) (355).

SV40 is enclosed within an ∼45 nm capsid composed of two layers, the outer formed of VP1 shielding an inner hydrophobic layer composed of VP2 and VP3. The VP2/3 layer encapsulates an ∼5 kbp circular double-stranded DNA genome, packaged into chromatin throughout its lifecycle (356, 357). Upon endocytosis, SV40-containing endosomes are targeted to the ER, where disulfide isomerase proteins reduce bonds formed between VP1 coat proteins, triggering partial capsid disassembly and exposure of the VP2/3 layer (358, 359, 360). The partially uncoated capsid is subsequently trafficked into the cytoplasm *via* the ER-associated degradation pathway, through direct interactions with DNAJ family chaperones, which in turn recruit cytoplasmic HSC70 and HSP105 to facilitate extraction (361, 362, 363). Within the cytoplasm, further remodeling is mediated by the dynein cargo adapters BICD2 and BICDR1, which bind exposed VP3 *via* coiled-coil domains. These cargo adapters serve dual functions: first, they promote additional capsid disassembly, facilitating the shedding of residual VP1 pentamers, and second, they tether the resultant vRNP complex to the microtubule network, facilitating cytosolic transport toward the NE (364). VP1 coat shedding exposes the inner VP2/3 layer, with VP3 shown to harbor an essential classical NLS that recruits KAPα•KAPβ1 for NPC translocation (Fig. 5*H*) (365). However, fluorescence microscopy tracking labeled SV40 DNA and VP2/3 proteins shows that the NLS-containing coat proteins do not accumulate in the nucleus, suggesting that additional vRNP remodeling may precede NPC translocation (366).

Another prominent example is IAV, a member of the Orthomyxoviridae family of enveloped, segmented, negative-sense single-stranded RNA viruses (292). Each virion contains eight distinct RNA segments, individually coated with multiple NPs at a ratio of ∼1 NP per 24 nucleotides. Each RNA also binds a single copy of the heterotrimeric polymerase complex composed of PB1, PB2, and PA, to form discrete vRNPs. Following endocytosis, these vRNPs are released from endosomes through the coordinated action of hemagglutinin and the M2 proton channel, both located on the virion’s surface (367, 368). Acidification of the capsid interior by M2 triggers dissociation of the matrix protein M1, liberating the vRNPs, which are then released into the cytoplasm *via* hemagglutinin-mediated fusion of the viral and endosomal membranes. In the cytoplasm, translocation across the NPC has been shown to depend on canonical RAN(GTP)-mediated nuclear import (369). vRNPs associate with karyopherins *via* a suite of classical and nonclassical NLSs encoded by NP, PB1, and PB2 (370, 371, 372). Although each vRNP delivers a single polymerase trimer, efficient infection requires additional polymerase subunits be imported separately. Fluorescence microscopy analyses show that PB1 and PA are cotransported, whereas PB2 enters separately, with deletion of the PB2 NLS leading to cytoplasmic accumulation of defective polymerase complexes (373). However, bulk vRNP import is primarily mediated by NP, which contains an essential nonclassical NLS at its N terminus, along with a downstream classical bipartite NLS (374, 375, 376, 377). Structures of *in vitro*–assembled vRNPs demonstrate that the N-terminal nonclassical NLS is exposed, whereas the downstream classical NLS is occluded (378, 379, 380). However, tissue-culture influenza infection models indicate that both NLSs are essential (381). Curiously, mutating the downstream classical NLS disrupts NP localization within the nucleus, suggesting an unexpected role for this signal later in the viral lifecycle. Nevertheless, questions remain regarding the stochastic nature of IAV import, with single-molecule fluorescence *in situ* hybridization studies suggesting that, at minimum, pairs of distinct vRNPs are coimported before dissociating in the nucleus (382).

### NPC-independent nuclear entry

The majority of viruses access the nucleus through the NPC, underscoring its vulnerability. However, a subset bypasses it entirely by exploiting either mitotic NE breakdown or by inducing structural defects in the NE and lamina to facilitate genome entry. The best understood examples are the *Papillomaviridae*, in particular HPV, which consists of an ∼55 nm icosahedral capsid encasing an ∼8 kbp circular double-stranded DNA genome (383). HPV is unable to infect quiescent cells and instead coordinates nuclear egress around the host’s mitotic cycle. Once internalized, endosomal acidification mediated by the vacuolar ATPase proton pump induces conformational changes at the major capsid protein L1, leading to separation from the minor capsid protein L2, which remains bound to the vDNA (384, 385). The L2•vDNA complex is further modified by N-terminal cleavage at L2, mediated by the protease furin, which permits retrograde trafficking into the *trans*-Golgi network (386). Challenging the notion that capsid remodeling is essential, recent studies have indicated that complete furin cleavage is uncommon *in vivo* and that some HPV capsids, such as HPV-16, remain largely intact throughout their lifecycle (Fig. 5*I*) (387, 388). Regardless, during interphase, the L2•vDNA complex remains sequestered within vesicles in the perinuclear space, shielding the viral genome from host nucleases (389, 390, 391). At the onset of mitosis, NE breakdown exposes the L2•vDNA complex to the cytoplasm, where the mitotic kinases CDK1 and polo-like kinase 1 transiently phosphorylate L2, priming its chromatin-binding domain (392, 393, 394). This modification promotes association with mitotic chromosomes and anchors the L2•vDNA complex inside the nascent nucleus after the NE reforms.

Finally, members of the Parvoviridae family (*e.g.*, minute virus of mice) have evolved an active NE disruption strategy. In digitonin-permeabilized HeLa cells, minute virus of mice induces transient NE permeability, demonstrated by the nuclear accumulation of large dextrans >150 kDa in mass (395, 396). This permeabilization correlates with the nuclear mislocalization of caspase-3, an apoptotic protease, resulting in small NE ruptures and cleavage of the nuclear lamina constituent lamin B2, ∼2 h after infection (Fig. 5*J*).

## Targeting karyopherins: A viral strategy to block nucleocytoplasmic transport

Antiviral responses rely on JAK–STAT and NF-κB-mediated import of transcription factors, which drive downstream expression of ISGs and proinflammatory cytokines upon viral detection. Briefly, extracellular activation of the JAK–STAT1 signaling cascade induces cytoplasmic phosphorylation of STAT1 and STAT2, triggering homodimerization or heterodimerization (397, 398). Tyrosine710-phosphorylated STAT1 (pY-STAT1) import depends on specific KAPα isoforms, including KAPα1, KAPα5, and KAPα6 (399, 400). In the nucleus, pY-STAT1 binds DNA and promotes the expression of downstream ISGs (401). Highlighting the nonredundant role of STAT1 signaling, mutants defective in nuclear import show reduced ISG expression (400). NF-κB signaling is likewise regulated by the interplay between post-translational modifications and nuclear import. NF-κB dimers, typically p50/p65, are sequestered in the cytoplasm by the inhibitor protein IκB (402, 403). Upon stimulation, the IκB kinase complex phosphorylates IκB, targeting it for degradation and releasing NF-κB dimers, which translocate into the nucleus *via* the classical import pathway. Accordingly, KAPα silencing reduces nuclear accumulation of p65 (287). Unsurprisingly, given the central role of nuclear import in transmitting innate immune signals, many viruses have evolved virulence factors that directly target karyopherins to promote propagation.

### α-Karyopherin competition

**Figure 6:**
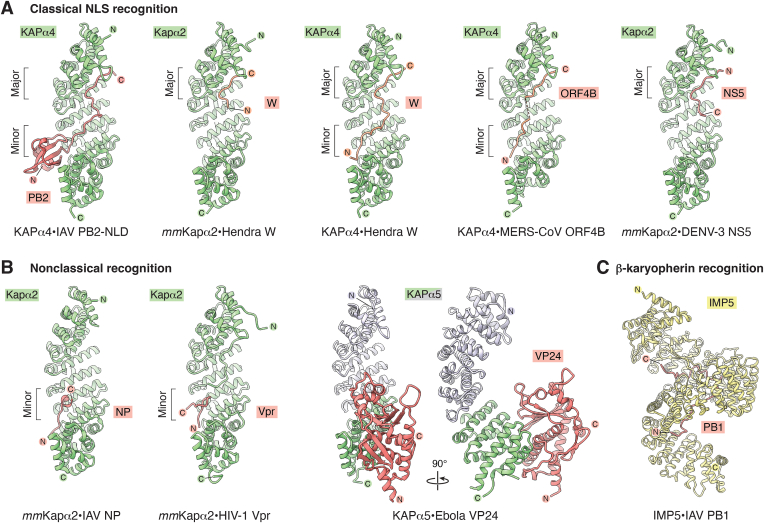
**Viral disruption of karyopherin function.***A*, crystal structures of α-karyopherin cargo adapters in complex with viral proteins containing classical nuclear localization signals (NLSs) highlight their strategic use of the classical nuclear import pathway to either force entry into the nucleus or competitively inhibit the import of host cargoes, including key effectors of the innate immune response and other nuclear regulators: influenza A virus (IAV) PB2 in complex with KAPα4 (PDB ID: 4UAE) (371); Hendra virus W protein in complex with *mm*Kapα2 (PDB ID: 6BW1) and KAPα4 (PDB ID: 6BW9) (406); Middle East respiratory syndrome coronavirus (MERS-CoV) ORF4B in complex with KAPα4 (PDB ID: 7RFY) (410); Dengue virus-3 (DENV-3) NS5 in complex with *mm*Kapα2 (PDB ID: 5FC8) (428). *B*, crystal structures of viral proteins exhibiting nonclassical interactions with α-karyopherin cargo adapters: IAV NP in complex with *mm*Kapα2 (PDB ID: 4ZDU) (370); HIV-1 Vpr in complex with *mm*Kapα2 (PDB ID: 5B56) (413); ebola virus VP24 in complex with a C-terminal fragment of KAPα5 (PDB ID: 4U2X) (415), N-terminal region sourced from the AlphaFold3 repository (*gray*) (502, 503). *C*, crystal structure of the nonclassical β-karyopherin NLS from IAV PB1 in complex with IMP5 (PDB ID: 9IM6) (372). PDB, Protein Data Bank.

Several viral proteins act as molecular “plugs” that inhibit host nuclear import by targeting the classical import pathway, specifically occupying the canonical NLS-binding sites on α-karyopherins (see the *Classical NLS recognition by α-karyopherins* section). The PB2 subunit of IAV polymerase contains a C-terminal domain harboring a bipartite NLS, engaging both the major and minor grooves, exhibiting low-nanomolar binding affinity with a mild preference for KAPα4 and KAPα6 isoforms (Fig. 6*A*). In addition, host-adaptive PB2 mutations (D701N, E627K) enhance affinity and broaden α-karyopherin isoform specificity, enabling them to outcompete host proteins for nuclear access (371, 404). Similarly, nonstructural *Henipavirus* W proteins from zoonotic Hendra and Nipah viruses preferentially bind KAPα4, exhibiting single-digit nanomolar affinity of two orders of magnitude tighter than observed for alternative isoforms (405). Structural studies show that W•KAPα4 forms an extended interface compared with other KAPα isoforms (Fig. 6*A*) (406). Functionally, nuclear localization of W proteins has been shown to suppress type-II IFN expression, through high-affinity α-karyopherin interactions that sequester the cellular pool of import adapters, reducing nuclear import capacity (405, 407). For example, W proteins trap 14-3-3 regulatory proteins in the nucleus, resulting in reduced NF-κB phosphorylation and the subsequent sequestration of this highly mobile transcription factor in the nuclear compartment (408).

Likewise, the vaccinia virus E3 ligase adapter A55 selectively targets KAPα2 to inhibit NF-κB signaling (409). A55 binds KAPα2 *via* its Kelch domain, and its overexpression suppresses p65 phosphorylation and nuclear translocation in ∼60% of tumor necrosis factor-α–stimulated cells. Middle East respiratory syndrome coronavirus expresses ORF4b, which encodes a bipartite NLS that preferentially binds KAPα4 (Fig. 6*A*) (410). Mutations in this region abolish nuclear localization of ORF4b in both Huh-7 and cultured human airway epithelial (Calu-3) cells, impairing its ability to block NF-κB nuclear import (411). Finally, HIV-1 accessory viral protein R (Vpr) was initially thought to contribute to viral nuclear import (see the *Passage of intact capsids across the NPC* section), yet primarily functions to evade host immune responses. In macrophages, Vpr antagonizes IFN signaling by inhibiting the nuclear localization of IRF3 and NF-κB, attenuating the upstream activity of cyclic GMP–AMP synthase and the stimulator of interferon genes, which detects cytosolic DNA as a marker of viral invasion (412). Vpr was found to interact with multiple α-karyopherin isoforms in coimmunoprecipitation experiments (412), binding *via* a noncanonical NLS at the minor NLS-binding groove (Fig. 6*B*) (413).

Beyond targeting the NLS-binding groove, some viral proteins engage remote, noncanonical α-karyopherin surfaces. For example, in Ebola virus–infected cells, IFN-induced nuclear accumulation of pY-STAT1 is blocked, a phenotype attributed to VP24-mediated disruption of the KAPα1•pY-STAT1 import complex (414). VP24 binds the C-terminal, convex face formed by armadillo repeats 7 to 10 of KAPα1, KAPα5, and KAPα6, an interface that overlaps with the atypical dimeric STAT1-binding site (Fig. 6*B*) (415). Structure-guided mutations that disrupt VP24•α-karyopherin interactions restore STAT1 nuclear import *in vivo*, validating the functional significance of this noncanonical binding mode (416). Likewise, HBV polymerase (Pol) has emerged as a virulence factor that interferes with STAT nuclear import. *In vivo* expression of Pol correlates with KAPα1-mediated cytoplasmic retention of STAT1 and STAT2, as shown by immunofluorescence and cellular fractionation (417). Biochemical analyses further reveal that HBV Pol binds KAPα1 *via* an overlapping site to the STAT1/2•KAPα1 interface. These findings illustrate a common viral strategy, whereby viral proteins mimic or disrupt NLS•α-karyopherin interactions to subvert nuclear import and suppress host immune signaling.

The Togaviridae (*e.g.*, Venezuelan equine encephalitis virus [VEEV], Chikungunya virus [CHIKV], Semliki Forest virus; Sindbis virus; and Mayaro virus) constitute a family of enveloped, single-stranded, positive-sense RNA viruses that replicate exclusively in the cytoplasm and manipulate nucleocytoplasmic transport *via* incompletely understood mechanisms (418). VEEV exploits bidirectional karyopherin transport to impair both transcriptional activity and NPC function (419). Specifically, VEEV produces capsid and nonstructural protein 2 (nsP2), each harboring independent NLS and NES motifs. nsP2 undergoes nucleocytoplasmic shuttling *via* KAPα1- and CRM1-dependent interactions, with the nuclear fraction inhibiting transcription (420). Functionally, the Mayaro virus nsP2 homolog reduces cellular levels of RNA polymerase II and the transcription initiation factor TFIIE2, with mutation of a conserved V-loop region preventing transcription inhibition in the CHIKV homolog (421, 422). Whereas nsP2 activity is conserved throughout Togaviridae, only VEEV capsid and not its CHIKV counterpart contains an atypical overlapping NLS–NES region that simultaneously recruits KAPα•KAPβ1 and CRM1 to assemble a stable heterotetramer, which accumulates at the central transport channel of the NPC (423). This unusual localization confers a nuclear import defect in human cells that is not recapitulated in the mosquito vector, indicating that VEEV exploits species-specific differences in the mobile karyopherin transport machinery (424).

### Virulence factors targeting β-karyopherins

A growing body of biochemical work shows that viral virulence factors co-opt β-karyopherins by masquerading as nuclear cargo to access the nucleus. For example, the HPV-16 minor capsid protein L2, which remains bound to the viral genome throughout its lifecycle (see the *NPC-independent nuclear entry* section), must be imported to the nucleus during formation of nascent virions. Notably, HPV-16 prioritizes nuclear entry of L2 by encoding two functionally redundant karyopherin-binding motifs (425, 426). The first classical NLS engages α-karyopherins, whereas the second, a nonclassical NLS, interacts directly with the β-karyopherins, KAPβ2 and TRANSPORTIN-3. Surprisingly, *in vivo* functional assays using GFP-L2 fusion proteins have demonstrated that efficient nuclear localization of L2 requires the coordinated activity of both signals, with disruption of either pathway impairing nuclear accumulation. A similar strategy of redundant NLS distribution is observed throughout Flaviviridae NS5 proteins, which encode two distinct NLSs within an unstructured linker region between globular domains, targeting α-karyopherins or KAPβ1 (427, 428) (Fig. 6*A*). Curiously, another viral factor, NS3, modulates NS5 import in a phosphorylation-dependent fashion by abrogating NS3•NS5 interactions (429). Functionally, NS3 competes with the KAPβ1 NLS, but not the classical KAPα NLS, implying that like HPV-16 L2, both NLSs are required for efficient nuclear import.

During IAV infection, the viral RNA genome is unpackaged into vRNPs containing NP and the PB1•PB2•PA polymerase. Notably, KAPβ2 binds the M1 coat protein, which is gradually shed in the cytoplasm prior to nuclear import, with RNAi screens revealing this interaction is essential (430). Mechanistically, KAPβ2 recognizes a PY-NLS located near the N terminus of M1, which may promote its dissociation from cytoplasmic vRNPs. IAV uncoating exposes both classical and nonclassical NLSs on the NP and polymerase subunits, enabling their recognition by α-karyopherins and promoting nuclear import (Fig. 6*B*, see the *Partial capsid disassembly and import of subviral components* section). Another example of viral exploitation of KAPβ2 is the human cytomegalovirus (HCMV) late viral transcription factor UL79 (431), which is essential for viral replication and functions in coordination with UL87 to reprogram viral protein expression to favor virion production (432, 433, 434). Fluorescence microscopy of COS-7 and human embryonic kidney 293T cells expressing labeled UL79 reveals primarily nuclear localization, with coexpression of the KAPβ2 inhibitor peptide M9M inhibiting nuclear accumulation *via* competition with an N-terminal PY-NLS (431). In addition, IAV PB1, a core component of the viral RNA–polymerase complex, undergoes conformational transition from a β-ribbon to an unstructured nonclassical NLS (Fig. 6*C*). Curiously, this atypical NLS spans both the N- and C-terminal arches of its nuclear import karyopherin, IMP5 (372).

## Viral factors targeting RAE1•NUP98

**Figure 7:**
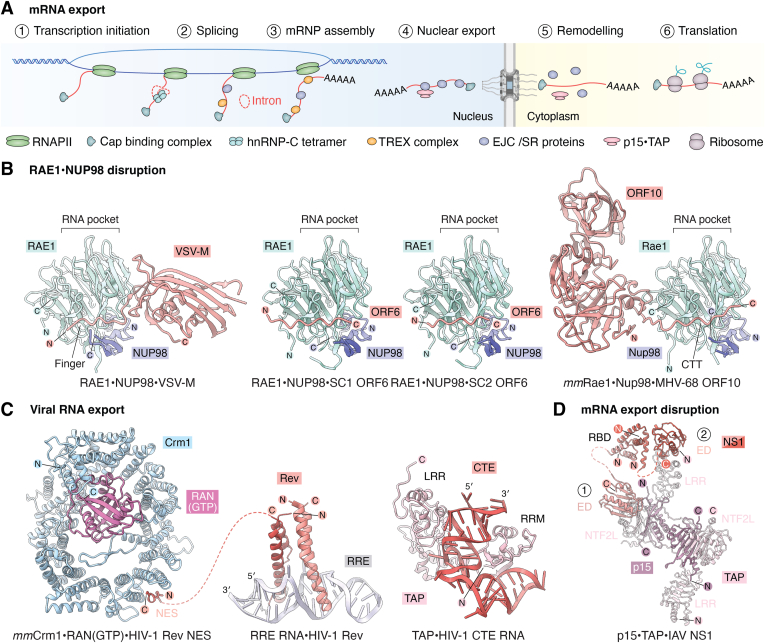
**Viral interference of mRNA export.***A*, schematic representation of mRNP assembly and export. In the nucleus: mRNA is transcribed by RNA polymerase II, and a 7-methylguanylate cap is deposited at the 5′ end of the transcript recruiting the cap-binding complex (CBC). Nascent RNA is then compacted by heterogeneous nuclear ribonucleoprotein (hnRNP)-C heterotetramers, which are required for efficient splicing. Following splicing exon junction complexes (EJCs) are deposited downstream from splice sites and the TRanscription EXport (TREX) complex is loaded. The mRNP is then “licensed” for export to the cytoplasm by the DEAD-box helicase, UAP56, which binds TREX and facilitates loading of the p15•TAP export factors to produce an mRNP. The mRNP is subsequently exported through the NPC and remodeled at the cytoplasmic face, where the helicase activity of the DEAD-box helicase DDX19 removes p15•TAP preventing futile import of mRNA into the nucleus. *B*, crystal structures of viral virulence factors bound at the RNA binding pocket of the cytoplasmic filament nucleoporin complex RAE1•NUP98: vesicular stomatitis virus (VSV)-M protein in complex with RAE1•NUP98^GLEBS^ (PDB ID: 4OWR) (444); severe acute respiratory syndrome coronavirus 1 (SARS-CoV1) ORF6 in complex with RAE1•NUP98^GLEBS^ (PDB ID: 7VPG); SARS-CoV2 ORF6 in complex with RAE1•NUP98^GLEBS^ (PDB ID: 7VPH) (452); murine γ-herpesvirus (MHV)-68 ORF10 in complex with *mm*Rae1•Nup98 ^GLEBS^ (PDB ID: 7BYF) (458). *C*, crystal structures of viral RNA elements illustrate how viruses hijack host nuclear export machinery to selectively promote the export of viral mRNAs: HIV-1 Rev nuclear export signal (NES) in complex with *mm*Crm1•RAN(GTP) (PDB ID: 3NBZ) (202); linked to a C-terminal region of HIV-1 Rev in complex with Rev response element (RRE) RNA (PDB ID: 4PM1) (468); HIV-1 constitutive transport element (CTE) in complex with nuclear export factor TAP (PDB ID: 3RW6) (251). *D*, crystal structure of functionally disruptive influenza A virus (IAV) NS1 in complex with the nuclear export factor p15•TAP (PDB ID: 6E5U) (485). NS1 homodimerizes to permit C-terminal effector domain (ED) interactions with the LLR and NTF2L domains of TAP. mRNP, messenger ribonucleoprotein; NS1, nonstructural protein 1.

The cytoplasmic filament nucleoporin RAE1 interacts with NUP98 through its GLEBS domain, forming a flexibly tethered RNA-binding platform involved in mRNA export (Fig. 7*A*; see the *mRNA export* section), although its precise function remains uncharacterized (88, 265). Disruption of this complex, through overexpression of decoy NUP98 GLEBS, causes nuclear accumulation of poly(A)^+^ RNA (435). Reflecting its central importance, diverse viral pathogens have evolved proteins targeting RAE1•NUP98, which impair host mRNA export and surprisingly also interfere with the nuclear import of immune signaling molecules (436, 437, 438, 439, 440).

This mode of action was first encountered in vesicular stomatitis virus (VSV), which primarily infects livestock and infrequently humans (441). A hallmark of VSV infection is the global suppression of host mRNA translation, whereas viral protein synthesis proceeds unimpeded (442). During infection, the VSV matrix protein (M) localizes at the NPC, causing nuclear retention of host mRNA (437, 443). Structurally, VSV-M binds to RAE1•NUP98 *via* its N-terminal “finger” extension, which inserts an essential methionine (M51) residue into a deep RNA-binding pocket on the surface of RAE1 (Fig. 7*B*) (444). *In vitro* RNA-binding assays have indicated that VSV-M outcompetes RNA binding to RAE1•NUP98 (444), whereas *in vivo* experiments have demonstrated that wildtype VSV-M, but not the disruptive M51R mutant, inhibits nuclear mRNA export and STAT import, suppressing the JAK–STAT signaling pathway (436, 445). Accordingly, *X. laevis* oocytes injected with recombinant VSV-M exhibit reduced nuclear import rates *via* the classical KAPα•KAPβ1-dependent pathway (436). However, they do not fully recapitulate the import defect observed in VSV-infected cells, suggesting the presence of confounding factors (437, 446).

SARS-CoV2, the causative agent of coronavirus disease 2019, has caused an unprecedented global pandemic, with ORF6 emerging as a potent virulence factor that disrupts nucleocytoplasmic transport (438, 447). A conserved ORF6 homolog in the related *Sarbecovirus* SARS-CoV1 antagonizes host innate immunity and has been proposed to block STAT1 nuclear import by selectively sequestering KAPα2, an isoform not typically implicated in STAT trafficking (448, 449). Recent studies suggest that SARS-CoV2 ORF6 also binds KAPα2 (438, 450), but whether *Sarbecovirus* ORF6 proteins share a conserved mechanism targeting α-karyopherins during immune evasion remains unclear. Beyond karyopherins, proteomic studies show that SARS-CoV2 ORF6 interacts with RAE1 and NUP98 (451). ORF6 engages the RNA-binding pocket of RAE1 *via* a methionine residue located in a C-terminal unstructured region (Fig. 7*B*), with mutation of this residue (M58R) disrupting RAE1•NUP98 binding and restoring STAT1 nuclear import (438, 452, 453). Consistent with inhibition of mRNA export, both SARS-CoV2 infection and ORF6 expression cause nuclear mRNA accumulation, phenocopying the effect of VSV-M. This effect can be reversed by RAE1 overexpression, which likely sequesters ORF6 apart from the NPC (439). ORF6 is a short, primarily unstructured 61-residue polypeptide encoding an N-terminal hydrophobic helix that promotes self-association (454, 455). Homo-oligomerization was demonstrated *in vitro*, where ORF6 forms protofilaments on lipid membranes, with assembly favored under physiological temperature and polarity conditions (454). However, the mechanistic link between ORF6 self-association and virulence, notably the disruption of bidirectional nucleocytoplasmic transport, remains a key open question.

Kaposi’s sarcoma–associated herpesvirus (KSHV), a large DNA virus implicated in several malignancies including Kaposi sarcoma and primary effusion lymphomas, also disrupts host mRNA export (456). KSHV infection or expression of a single virulence factor, ORF10, induces nuclear accumulation of poly(A)^+^ RNA (457). RNA-Seq analysis demonstrates that ∼24% of cytosolic transcripts (2971 of 12,181) are significantly reduced in ORF10-transfected cells, a defect rescued by either NUP98 knockdown or RAE1 overexpression, with both acting to draw the virulence factor away from the NPC (458). Notably, retained transcripts shared common 3′ UTR features, suggesting transcript-selective mRNA export inhibition. Structurally, ORF10 encodes a C-terminal tail, which engages an extended interface on RAE1 centered around its RNA-binding pocket (Fig. 7*B*). However, unlike VSV-M or ORF6, the RAE1•NUP98•ORF10 complex retains RNA-binding capacity, indicating that ORF10 overrides RAE1’s native specificity and contributes its own RNA-binding selectivity to support the export of transcripts promoting infection. Supporting this model, the ORF10•RAE1 interaction is necessary for the expression of late viral genes involved in virion production, suggesting that ORF10 specifically redirects host mRNA export to favor viral gene expression.

Finally, IAV infection broadly induces nuclear poly(A)^+^ RNA accumulation in Madin–Darby canine kidney cells. The viral nonstructural protein 1 (NS1) forms inhibitory complexes with several mRNA export factors, including p15•TAP and RAE1, as shown by *in vitro* pull-down assays (459). Overexpression of RAE1, p15, or TAP partially rescues the export defect in NS1-expressing cells, suggesting that mRNA export is impaired by sequestering mRNP export complexes apart from the NPC. Crucially, NS1 expression also reduces RAE1•NUP98 complex levels during infection, a biologically significant effect, as RAE1^+^/^−^ or NUP98^+^/^−^ cells show increased sensitivity to IAV infection.

## Viral suppression of host mRNA export and redirection toward viral RNA transport

In host cells, nascent mRNAs are packaged into mRNPs that are exported through the NPC (see the *mRNA export* section). Viruses replicating in the nucleus commonly subvert the host’s mRNA export machinery, redirecting it toward the export of viral transcripts, while dampening the export of antiviral host factors.

### Expediting viral mRNA export *via* the CRM1-dependent pathway

Retroviruses such as HIV-1 present a unique regulatory challenge, as their genome is integrated into host chromosomes and consequently transcribed in the nucleus. To expand their coding capacity, they exploit differential splicing, with spliced transcripts typically encoding regulatory proteins, whereas unspliced or partially processed viral mRNAs generate structural proteins. However, under typical conditions, intron-containing transcripts are retained and degraded by the host’s mRNA export quality control machinery (460, 461). To circumvent this, HIV-1 expresses the regulator of expression of virion protein (Rev), which shuttles into the nucleus and binds specifically to intron-containing viral mRNAs (462, 463). A representative example is the Rev-dependent cytoplasmic accumulation of unspliceable HIV-1 envelope transcripts, indicating that Rev functions to promote nuclear export, rather than splicing (462). Functionally, Rev forms oligomers that bind a structured RNA element within the viral transcript known as the Rev response element (Fig. 7*C*) (462, 464, 465, 466). This interaction induces conformational changes in the RNA that modulate viral replication efficiency (467, 468). To facilitate nuclear export, Rev bypasses the mRNA export pathway entirely and instead encodes a leucine-rich NES that recruits CRM1, enabling the active export of intron-containing viral RNAs *via* the RAN-dependent karyopherin pathway (Fig. 7*C*) (202, 469, 470). Similarly, IAV also exports its nascent vRNPs *via* a CRM1-dependent mechanism (284).

### Viral proteins impairing host mRNA export

HSV-1 replicates within the nucleus and has evolved specialized factors promoting the export of viral transcripts, while concurrently suppressing host mRNA export. Infected cell protein 27 (ICP27) is a regulatory HSV-1 protein with multiple functions in RNA transcription, processing, and export (471, 472, 473, 474). ICP27 is known to inhibit host pre-mRNA splicing by interacting with the SR protein kinase. During HSV-1 infection, pre-mRNA phosphorylation is suppressed, resulting in defective spliceosome assembly and transcriptional stalling, which can be rescued by supplementing ICP27-inhibited nuclear extracts with exogenous SR protein kinase (475). Concurrently, the unspliced or minimally spliced viral mRNA utilizes ICP27 to recruit the mRNA export factor ALYREF, a key component of the TREX complex, which in turn recruits EJCs that are typically deposited on spliced mRNAs (474, 476). Accordingly, ICP27 mutants unable to bind to ALYREF lead to defects in viral mRNA export and reduced viral propagation (477). The dual function of ICP27 ensures preferential export of viral mRNAs by redirecting the host’s bulk mRNA export machinery toward viral transcripts, while simultaneously inhibiting host mRNP assembly. This mechanism is conserved across Herpesviridae, for example, Epstein–Barr virus EB2 and KSHV ORF57 share an ICP27 homology domain that facilitates the recruitment of ALYREF and the p15•TAP export complex to unspliced viral mRNAs (237, 478, 479, 480, 481).

The disruption of host mRNA export by multifunctional viral proteins is a recurring strategy across diverse viruses. HCMV, a large double-stranded DNA virus, expresses UL69, which disrupts the activity of the DEAD-box RNA helicase UAP56, which is involved in nucleoplasmic mRNP export (482). Interestingly, HCMV also promotes host translation through two mechanisms: first, UL38 sustains constitutive mTORC1 kinase activity, maintaining the negative regulator 4EBP1 in an inactive, hyperphosphorylated state (483). Second, UL69 interacts with eIF4A and the poly(A)^+^-binding protein (PABPN1) to exclude hypophosphorylated 4EBP1 from the mRNA CBC, a process essential for the accumulation of late viral proteins during infection. The IAV nonstructural protein 1–binding protein interacts with viral mRNA transcripts for NS1 and M1, inducing nuclear host mRNA accumulation and promoting an alternative splicing event that switches transcription between M1 and M2 matrix protein isoforms (484). Nonstructural protein 1–binding protein also competes with IAV NS1 protein for binding to the mRNA export factor TAP (485) (Fig. 7*D*), promoting TAP recruitment to M2 mRNAs and facilitating their export *via* the TREX-2-dependent pathway (486). Finally, the adenoviral proteins E1B-55K and E4orf6 play a central role in reprogramming host mRNA export. Together, they form a complex with the mRNP export factor p15•TAP, promoting the selective nuclear retention of host transcripts and prioritizing the export and translation of viral structural mRNAs during late infection (487, 488, 489). Notably, the E1B-55K•E4orf6 complex functions as a Cullin-based E3 ubiquitin ligase, promoting the degradation of certain mRNA export factors, to further enhance viral RNA export efficiency (490, 491).

## Concluding remarks

Viral interference with nucleocytoplasmic transport represents a multifaceted strategy to subvert host defenses and promote replication, employing both subtle manipulations and irreversible disruption (summarized in Fig. 1). The NPC has emerged as a critical cellular vulnerability, targeted by a broad spectrum of viruses. These intracellular parasites deploy divergent strategies to subvert nucleocytoplasmic transport, including the proteolytic cleavage of nucleoporins and karyopherins, the expression of proteins that mimic host NLS–NES signals, and the sequestration of transport factors. These mechanisms collectively facilitate viral nuclear access, while obstructing the import of key immune effectors. This striking functional convergence, in which unrelated viruses have independently evolved to target the NPC, highlights its role not merely as a conduit for macromolecular exchange but as an active participant during host innate immune responses. Future work will involve leveraging recent developments in NPC structure and function to design a new wave of antiviral therapeutics, targeting infection prior to the hijacking of the nucleocytoplasmic transport machinery and interruption of the host’s innate immune response.

## Conflict of interest

The authors declare that they have no conflicts of interest with the contents of this article.
